# Supplementary material for: Positive Feedback Defines the Timing, Magnitude, and Robustness of Angiogenesis
Source: Cell Rep. 2019 Jun 11;27(11):3139–3151.e5. doi: 10.1016/j.celrep.2019.05.052 (PMC6581738; doi:10.1016/j.celrep.2019.05.052)
Supplement: Document S1. Figures S1–S4 and Table S1 [file mmc1.pdf]

Cell Reports, Volume *27*

Supplemental Information

Positive Feedback Defines the Timing, Magnitude, and Robustness of Angiogenesis

Donna J. Page, Raphael Thuret, Lakshmi Venkatraman, Tokiharu Takahashi, Katie Bentley, and Shane P. Herbert

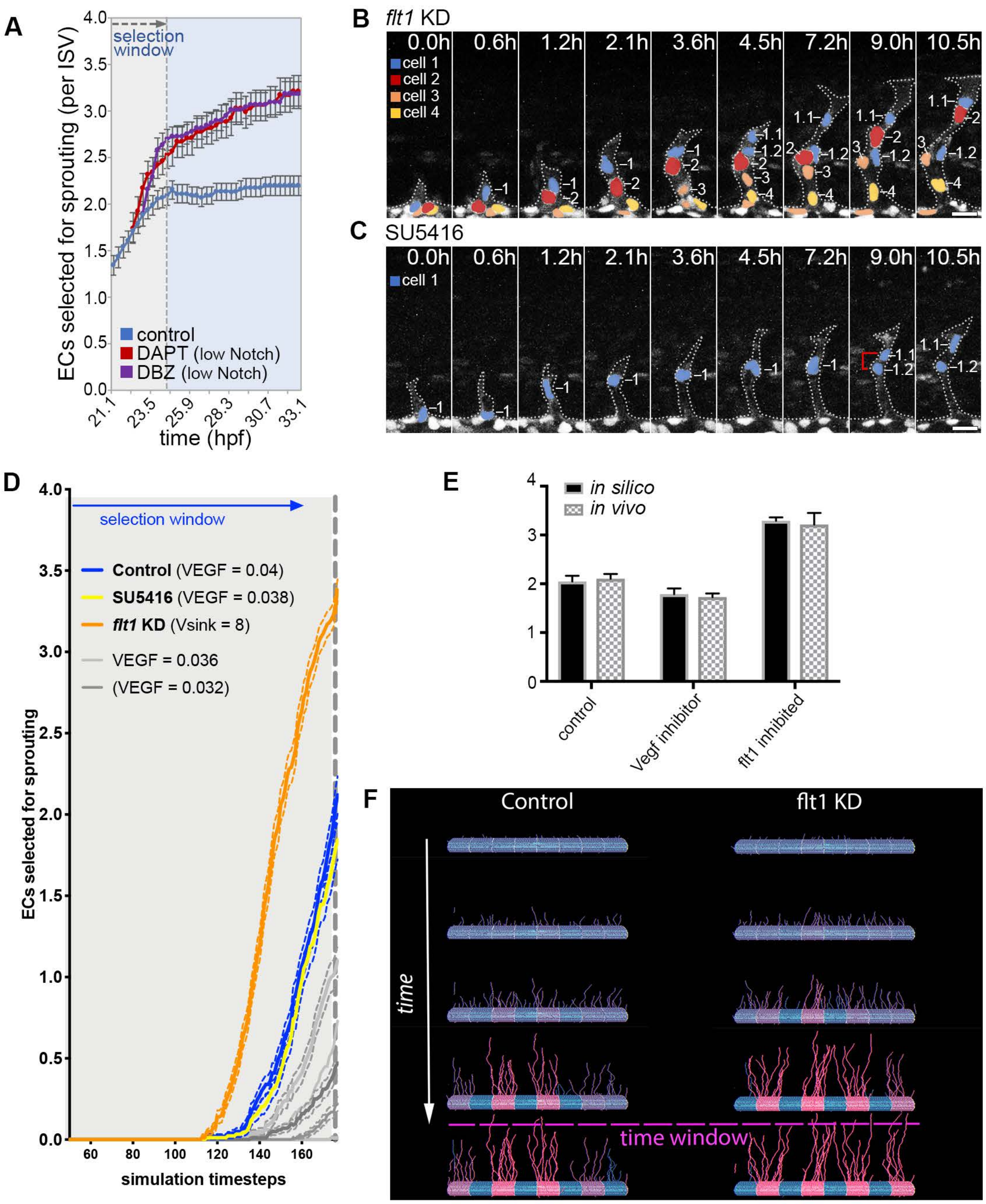

Figure S1. VEGFR signaling and positive-feedback define the magnitude and timing of tip EC selection. Related to Figure 2. (A) Quantification of the number of ECs selected to branch into ISVs of control, DAPT-treated and DBZ-treated embryos (*n* = 47 ISVs from 16 control, 35 ISVs from 8 DBZ-treated and 28 cells from 7 DAPT-treated embryos). (B and C) Time-lapse images of EC nuclei in sprouting ISVs of *flt1* KD (B) and SU5416-treated (C) *Tg(kdrl:nlsEGFP)*^{zf109} embryos from around 19 hours post-fertilization (hpf). Brackets indicate dividing cells. Nuclei are pseudocolored according to when the selected EC entered the ISV. (D) MSM modeling of the influence of differential Vegfr and *flt1* levels on the timing of EC activation via LI. By defining a selection window at approximately 175-time steps, the model accurately recapitulates the *in-vivo* behavior induced by Vegfr inhibition (decreased EC selection) and *flt1* KD (increased EC selection) in Figure 2C. (E) Quantification of the number of cells selected in the selection window both *in-silico* and *in-vivo.* (F) Example frames from one simulation run of a control simulation (Vsink=9) vs a *flt1* KD simulation (Vsink=8) showing by the end of the time window the control is actually less efficient, i.e. slower to select within the time window as the *flt1* KD selects all possible tip cells from the available pool of cells more rapidly. Frames taken at t=0,50,100,150,200. Scale bars, 25 μm.

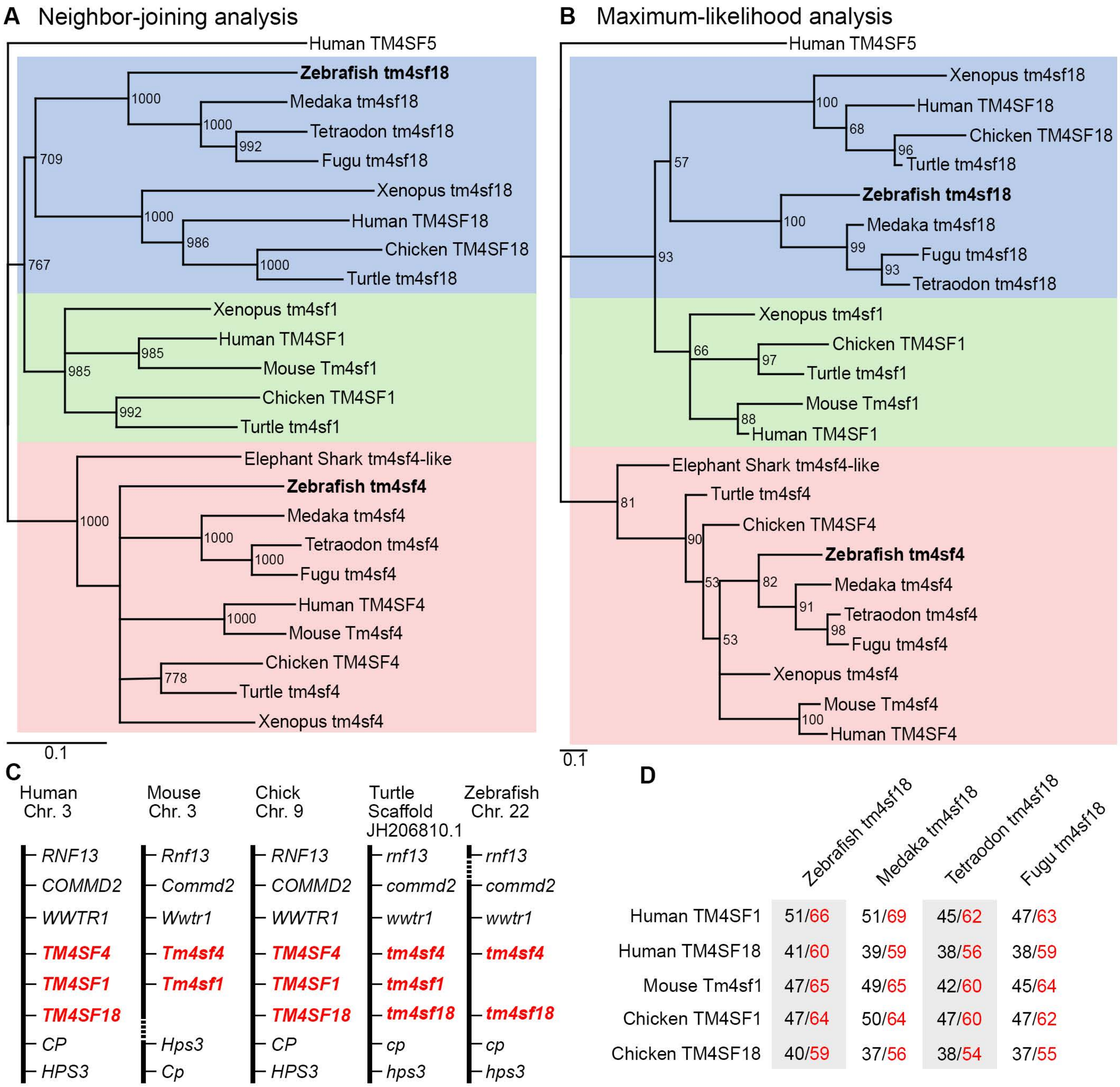

	Zebrafish tm4sf18	Medaka tm4sf18	Tetraodon tm4sf18	Fugu tm4sf18
Human TM4SF1	51/66	51/69	45/62	47/63
Human TM4SF18	41/60	39/59	38/56	38/59
Mouse Tm4sf1	47/65	49/65	42/60	45/64
Chicken TM4SF1	47/64	50/64	47/60	47/62
Chicken TM4SF18	40/59	37/56	38/54	37/55

Figure S2. Phylogenetic, synteny and protein analysis of the TM4SF family. Related to Figure 3. (A and B) Phylogenetic trees of vertebrate TM4SF1/4/18 protein family based on either neighbor-joining (NJ, A) or maximum-likelihood (ML, B) analyses. Human TM4SF5 protein sequence was defined as the out-group. Branch lengths are proportional to evolutionary distance corrected for multiple substitutions; the scale bar denotes 0.1 underlying amino acid substitutions per site. Figures on branches indicate robustness of each node (>50%), estimated from 1000 bootstrap replicates for NJ (A) and 100 replicates for ML (B). All nodes with a bootstrap support of less than 50% were collapsed to a polytomy. Based on separate analyses of all vertebrate TM4SF protein (data not shown), TM4SF1, 4 and 18 form a monophyletic group and likely share a common ancestor. Hence, TM4SF1 and TM4SF18 are the most related in the TM4SF family. (C) Synteny analysis of vertebrate *Tm4SF1/4/18* in the human, mouse, chick, turtle and zebrafish genome assemblies. Dashed lines represent breaks in synteny. *TM4SF1, 4* and *18* are closely located on the same chromosome, suggesting that they were generated by two tandem gene duplications at early stages of vertebrate evolution. The first of these gave rise to the *TM4SF4* and *TM4SF1/18* paralogous groups, and the second gave rise to the *TM4SF1* and *TM4SF18* paralogous groups. The *tm4sf1* subfamily appears to have subsequently been lost in the lineage leading to teleost fish. Similarly, the *Tm4sf18* subfamily appears to have subsequently been lost in mice. (D) Percentage BLAST sequence identity (black numbers) and sequence similarity (red numbers) between teleost fish (zebrafish, medaka, tetraodon and fugu) Tm4sf18 and human, mouse or chick TM4SF1/18. Following the loss of *tm4sf1*, the remaining Tm4sf18 in teleost fish consistently shares higher protein sequence identity/similarity with human and chicken TM4SF1 over TM4SF18. Hence, zebrafish Tm4sf18 appears to have acquired the function of Tm4sf1 and is the predominant teleost homologue of mammalian TM4SF1.

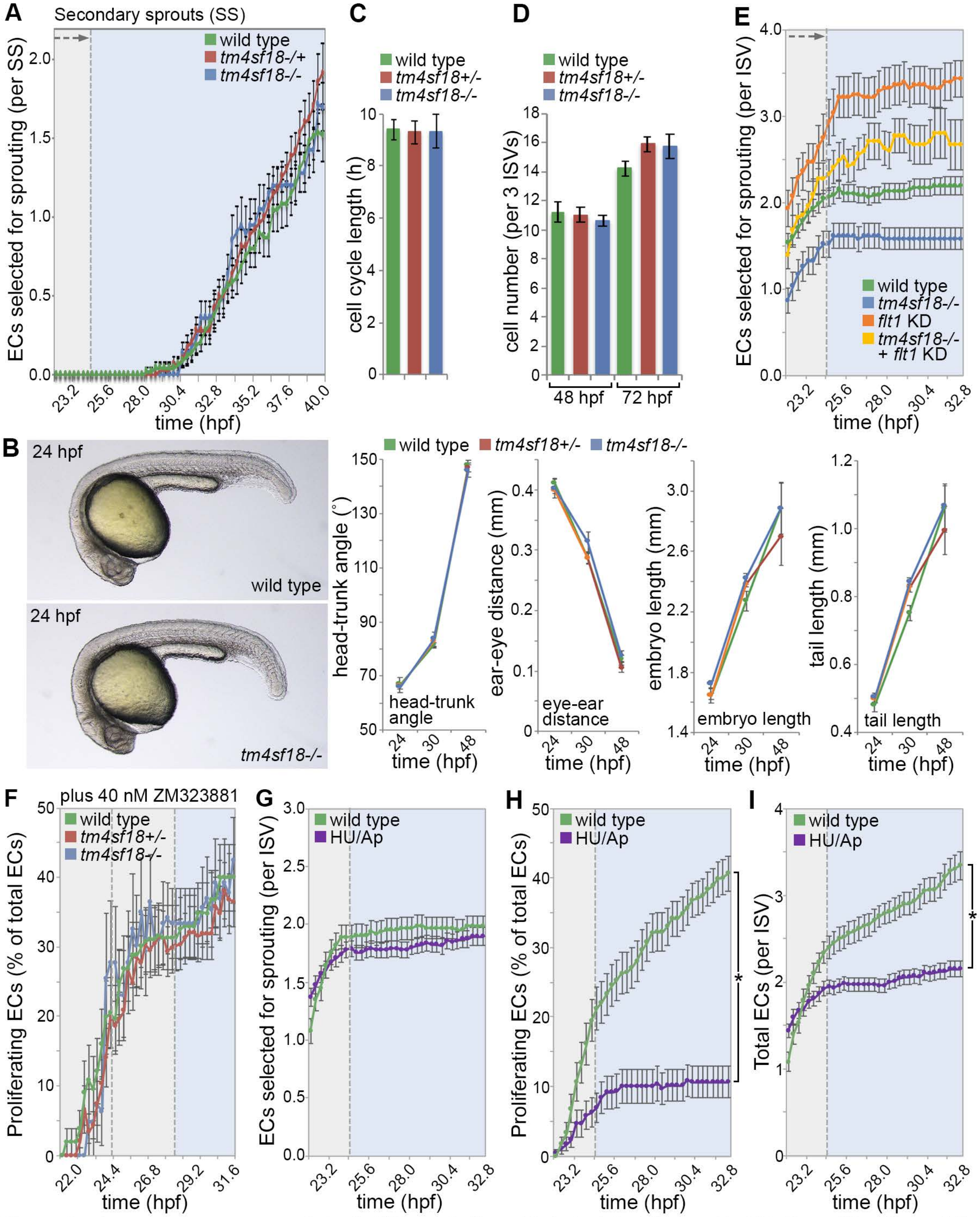

Figure S3. Phenotypic consequences of *tm4sf18* mutation. Related to Figure 5. (A) Quantification of the number of ECs selected to branch into venous secondary sprouts (SS) of WT, *tm4sf18*$^{+/-}$ and *tm4sf18*$^{-/-}$ embryos (*n* = at least 21 ISVs from 16 WT, 12 ISVs from 15 *tm4sf18*$^{+/-}$ and 10 ISVs from 8 *tm4sf18*$^{-/-}$ embryos at each time point). (B) Lateral views of 24 hpf WT and *tm4sf18*$^{-/-}$ homozygous mutant embryos and quantification of key developmental landmarks (*n* = 15 WT, 43 *tm4sf18*$^{+/-}$ and 12 *tm4sf18*$^{-/-}$ embryos at 24 hpf; *n* = 36 WT, 17 *tm4sf18*$^{+/-}$ and 8 *tm4sf18*$^{-/-}$ embryos at 30 hpf; *n* = 17 WT, 29 *tm4sf18*$^{+/-}$ and 18 *tm4sf18*$^{-/-}$ embryos at 48 hpf). (C to D) Quantification of cell cycle length (C; *n* = 29 divisions from 16 WT, 30 divisions from 15 *tm4sf18*$^{+/-}$ and 13 divisions from 8 *tm4sf18*$^{-/-}$ embryos) and ISV cell numbers at 48 hpf and 72 hpf in WT, *tm4sf18*$^{+/-}$ and *tm4sf18*$^{-/-}$ embryos (D; *n* = 18 WT, 38 *tm4sf18*$^{+/-}$ and 25 *tm4sf18*$^{-/-}$ embryos at 48 hpf; *n* = 18 WT, 39 *tm4sf18*$^{+/-}$ and 22 *tm4sf18*$^{-/-}$ embryos at 72 hpf). (E) Quantification of the number of ECs selected to branch into ISVs of WT, *tm4sf18*$^{-/-}$, *flt1* KD and combined *flt1* KD *tm4sf18*$^{-/-}$ embryos (*n* = 47 ISVs from 16 WT, 31 ISVs from 8 *tm4sf18*$^{-/-}$, 28 ISVs from 8 *flt1* KD and from 23 to 9 ISVs from 4 *tm4sf18*$^{-/-}$ *flt1* KD embryos). (F) Quantification of the percentage of ECs that undergo proliferation in WT, *tm4sf18*$^{+/-}$ and *tm4sf18*$^{-/-}$ embryos incubated with 40 nM ZM323881 from 18 hpf onward (*n* = 26 ISVs from 8 WT, 50 ISVs from 14 *tm4sf18*$^{+/-}$ and 21 ISVs from 6 *tm4sf18*$^{-/-}$ embryos). (G) Quantification of the number of ECs selected to branch into ISVs of WT or HU/Ap-treated embryos. (H) Quantification of the percentage of ECs that undergo proliferation in ISVs of WT or HU/Ap-treated embryos. (I) Quantification of the total number of ECs per ISV of WT or HU/Ap-treated embryos (*n* = 62 ISVs from 16 WT and 73 ISVs from 19 HU/Ap-treated embryos). Error bars: mean ± SEM. *$P<0.05$, two-way ANOVA or two-tailed t test.

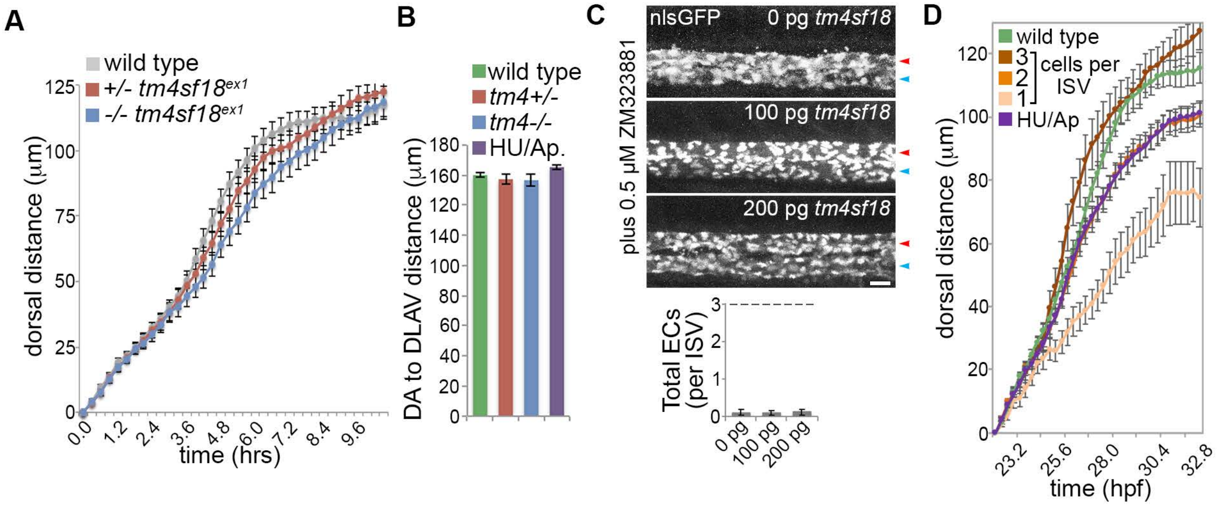

Figure S4. Phenotypic consequences of *tm4sf18* mutation on EC migration. Related to Figure 6. (A) Quantification of the dorsal movement of tip ECs in WT, $tm4sf18^{+/-}$ exon1 heterozygous mutant and $tm4sf18^{-/-}$ exon-1 homozygous mutant embryos. Mutation of the long (but not short) isoform of *tm4sf18* does not disrupt EC motility (*n* = 71 ISVs from 16 WT, 57 ISVs from 15 $tm4sf18^{+/-}$ exon-1 and 38 ISVs from 12 $tm4sf18^{-/-}$ exon-1 embryos). (B) Quantification of the length of any fully formed ISVs observed in WT, $tm4sf18^{+/-}$, $tm4sf18^{-/-}$ and HU/Ap-treated embryos. None of these perturbations affects the morphology of successfully formed ISVs (*n* = 48 ISVs from 16 WT, 45 ISVs from 15 $tm4sf18^{+/-}$, 24 ISVs from 8 $tm4sf18^{-/-}$ and 66 ISVs from 22 HU/Ap-treated embryos). (C) Lateral views of $Tg(kdrl:nlsEGFP)^{zf109}$ embryos at 30 hpf and quantification of total ECs per ISV following injection at the 1 cell stage with the indicated amount of *tm4sf18* mRNA and incubation with ZM323881 from 22 hpf. *tm4sf18* expression is insufficient to drive ISV sprouting in the absence of VEGFR activity. Red and blue arrowheads indicate the DA and PCV, respectively. Dashed line indicates the expected number of ECs per ISV at 30 hpf (*n* = 44 0 pg-injected, 44 100 pg-injected and 40 200 pg-injected embryos). (D) Quantification of the dorsal movement of tip ECs in ISV consisting of 1, 2 and 3 or more ECs and comparison with the motility of tip ECs in wild type and HU/Ap-treated embryos. Tip ECs in wild type embryos behave like those in ISVs with three cells whereas inhibition of proliferation generates tip ECs that behave like those in ISVs with only two cells, consistent with the average number of ECs per ISV in both of these situations (see Figure 5C; *n* = 71 ISVs from 16 WT and 89 ISVs from 22 HU/Ap-treated, as well as 17 ISVs with 3 cells, 53 ISVs with 2 cells and 16 ISVs with 1 cell from 22 embryos). Error bars: mean ± SEM. Scale bar, 25 µm.

Table S1. Primers used for quantitative real-time PCR (qPCR) and genotyping. Related to the STAR Methods.

Experiment	Species	Primer Name	Sequence
qPCR	zebrafish	*β actin* forward	5'-CGAGCTGTCTTCCCATCCA-3'
	zebrafish	*β actin* reverse	5'-TCACCAACGTAGCTGTCTTTCTG-3'
	zebrafish	*dll4* forward	5'-TGGCCAGTTATCCTGTCTCC-3'
	zebrafish	*dll4* reverse	5'-CTCACTGCATCCCTCCAGAC-3'
	zebrafish	*ef1α* forward	5'-CTGGAGGCCAGCTCAAACAT-3'
	zebrafish	*ef1α* reverse	5'-ATCAAGAAGAGTAGTACCGCTAGCATTAC-3'
	zebrafish	*flt4* forward	5'-CTGTCGGATTTGGATTGGGA-3'
	zebrafish	*flt4* reverse	5'-GGTGGACTCATAGAAAACCCATTC-3'
	zebrafish	*kdrl* forward	5'-ACTTTGAGTGGGAGTTTCATAAGGA-3'
	zebrafish	*kdrl* reverse	5'-TTGGACCGGTGTGGTGCTA-3'
	zebrafish	*tm4sf18* forward	5'-CTGGATACTGCTTCCTGATCTC-3'
	zebrafish	*tm4sf18* reverse	5'-CAAACAGATACCGTCCCTCAT-3'
	human	*GAPDH* forward	5'-TGCACCACCAACTGCTTAGC-3'
	human	*GAPDH* reverse	5'-GGCATGGACTGTGGTCATGAG-3'
	human	*TM4SF1* forward	5'-CTTCGTGTGGTTCTTTTCTG-3'
	human	*TM4SF1* reverse	5'-ATCGTTTGCCACAGTTTTC-3'
Genotyping	zebrafish	*tm4sf18* TALEN forward	5'-CTGTTTTCTCCCCCACACAC-3'
	zebrafish	*tm4sf18* TALEN reverse	5'-TACTCACAGCCAGACCACCA-3'
	zebrafish	*tm4sf18* CRISPR forward	5'- CATCAGTCTTTGCAGCGAGA -3'
	zebrafish	*tm4sf18* CRISPR reverse	5'- TGTAGCATATCCCAACACTCAC -3'
