# Supplementary material for: Positive Feedback Defines the Timing, Magnitude, and Robustness of Angiogenesis
Source: Cell Rep. 2019 Jun 11;27(11):3139–3151.e5. doi: 10.1016/j.celrep.2019.05.052 (PMC6581738; doi:10.1016/j.celrep.2019.05.052)
Supplement: Document S2. Article plus Supplemental Information [file mmc2.pdf]

Cell Reports

Article

Positive Feedback Defines the Timing, Magnitude, and Robustness of Angiogenesis

Graphical Abstract

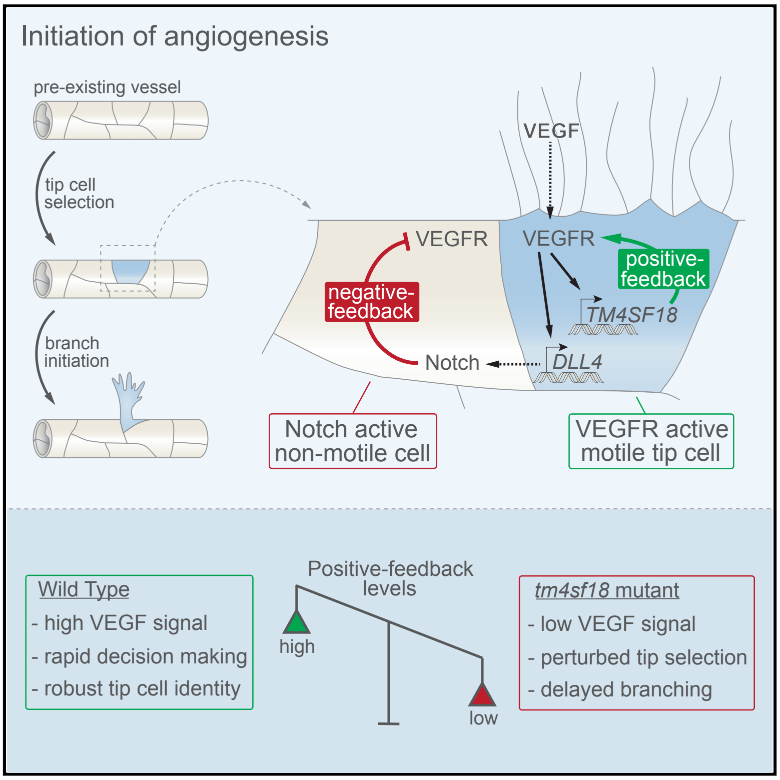

Highlights

- VEGF-driven positive feedback speeds up endothelial tip-stalk cell identity decisions
- Tm4sf18 is a positive-feedback modulator of VEGF signaling and angiogenesis *in vivo*
- Tm4sf18 shapes the timing, magnitude, and robustness of angiogenic identity decisions

Authors

Donna J. Page, Raphael Thuret, Lakshmi Venkatraman, Tokiharu Takahashi, Katie Bentley, Shane P. Herbert

Correspondence

katie.bentley@crick.ac.uk (K.B.),
shane.herbert@manchester.ac.uk (S.P.H.)

In Brief

In angiogenesis, tight control of endothelial tip cell selection and migration drives new blood vessel branching. Page et al. show that VEGF-signaling-mediated positive feedback shapes the timing, magnitude, and robustness of tip cell identity decisions. Moreover, they identify a key modulator of VEGF-mediated positive feedback, Tm4sf18, which temporally controls angiogenesis *in vivo*.

Page et al., 2019, Cell Reports *27*, 3139–3151
June 11, 2019 © 2019 The Authors.
https://doi.org/10.1016/j.celrep.2019.05.052

CellPress

OPEN ACCESS
CellPress

Positive Feedback Defines the Timing, Magnitude, and Robustness of Angiogenesis

Donna J. Page,[1,2,8] Raphael Thuret,[1,8] Lakshmi Venkatraman,[3,4] Tokiharu Takahashi,[1] Katie Bentley,[3,4,5,6,7,*] and Shane P. Herbert[1,9,*]
[1]Faculty of Biology, Medicine and Health, Michael Smith Building, University of Manchester, Oxford Road, Manchester M13 9PT, UK
[2]School of Healthcare Science, Manchester Metropolitan University, Manchester M1 5GD, UK
[3]Biomedical Engineering Department, Boston University, 610 Commonwealth Avenue, Boston, MA 02215, USA
[4]Immunology, Genetics and Pathology Department, University of Uppsala, 751 85 Uppsala, Sweden
[5]Center for Vascular Biology Research, Beth Israel Deaconess Medical Center, Harvard Medical School, Boston, MA 02215, USA
[6]Cellular Adaptive Behaviour Lab, The Francis Crick Institute, Midland Road, London NW1 1AT, UK
[7]Department of Informatics, Faculty of Natural and Mathematical Sciences, King's College London, Strand Campus, London WC2B 4BG, UK
[8]These authors contributed equally
[9]Lead Contact
*Correspondence: katie.bentley@crick.ac.uk (K.B.), shane.herbert@manchester.ac.uk (S.P.H.)
https://doi.org/10.1016/j.celrep.2019.05.052

SUMMARY

Angiogenesis is driven by the coordinated collective branching of specialized leading "tip" and trailing "stalk" endothelial cells (ECs). While Notch-regulated negative feedback suppresses excessive tip selection, roles for positive feedback in EC identity decisions remain unexplored. Here, by integrating computational modeling with *in vivo* experimentation, we reveal that positive feedback critically modulates the magnitude, timing, and robustness of angiogenic responses. *In silico* modeling predicts that positive-feedback-mediated amplification of VEGF signaling generates an ultrasensitive bistable switch that underpins quick and robust tip-stalk decisions. In agreement, we define a positive-feedback loop exhibiting these properties *in vivo*, whereby Vegf-induced expression of the atypical tetraspanin, *tm4sf18*, amplifies Vegf signaling to dictate the speed and robustness of EC selection for angiogenesis. Consequently, *tm4sf18* mutant zebrafish select fewer motile ECs and exhibit stunted hypocellular vessels with unstable tip identity that is severely perturbed by even subtle Vegfr attenuation. Hence, positive feedback spatiotemporally shapes the angiogenic switch to ultimately modulate vascular network topology.

INTRODUCTION

New blood vessel formation by the process of angiogenesis is critical for tissue development, homeostasis, and repair and is frequently dysregulated in disease (Carmeliet and Jain, 2011; Herbert and Stainier, 2011; Potente et al., 2011). Consequently, the tight control of angiogenesis is key to normal tissue and organ function. In particular, the behavior of sprouting endothelial cells (ECs) needs to be elegantly coordinated during new blood vessel branching (Carmeliet and Jain, 2011; Herbert and Stainier, 2011; Potente et al., 2011). For example, activation of vascular endothelial growth factor receptor (VEGFR)-2/-3 signaling by gradients of VEGF-A/-C ligand promotes selection of specialized "tip" ECs, which are highly motile and lead new blood vessel branches (Gerhardt et al., 2003; Ruhrberg et al., 2002) (Figure 1A). In contrast, "stalk" ECs experience less VEGFR signaling and trail behind tip cells. To prevent excessive sprouting, the induction of tip identity is repressed by DLL4-mediated Notch activation and lateral inhibition (LI) of non-sprouting EC populations (Benedito et al., 2012; Hellström et al., 2007; Hogan et al., 2009; Leslie et al., 2007; Lobov et al., 2007; Siekmann and Lawson, 2007; Suchting et al., 2007; Zarkada et al., 2015) (Figure 1A). During this process, VEGFR activation promotes upregulation of the Notch ligand DLL4 in emerging tip cells, which *trans*-activates Notch in neighboring cells. Elevated Notch activity promotes downregulation of VEGFR-2 and VEGFR-3 function, rendering laterally inhibited ECs less responsive to VEGF signal (Figure 1B) (Benedito et al., 2012; Hellström et al., 2007; Hogan et al., 2009; Leslie et al., 2007; Lobov et al., 2007; Siekmann and Lawson, 2007; Suchting et al., 2007; Zarkada et al., 2015). As such, DLL4-Notch signaling acts in a negative-feedback loop with VEGF that limits the number of ECs that acquire tip identity; consequently, loss of Notch signaling results in EC hyper-sprouting *in vivo*. Hence, negative-feedback-mediated competition of ECs for migratory status drives the coordinated collective movement of sprouting EC populations in angiogenesis.

Although negative feedback via DLL4-Notch plays well-established roles in the spatial control of VEGFR activity, the function and/or identity of positive-feedback modulators of VEGFR signaling and angiogenesis remains unclear. Positive-feedback loops commonly amplify signal outputs to shape the pattern, duration, and threshold of many signaling pathways. As such, positive feedback modulates key aspects of developmental signaling responses, such as their magnitude, robustness, and timing (Brandman and Meyer, 2008; Freeman, 2000). While it is clear that dynamic control of these aspects of EC decision making (such as the timing of tip-stalk selection) fundamentally

This is an open access article under the CC BY license (http://creativecommons.org/licenses/by/4.0/).

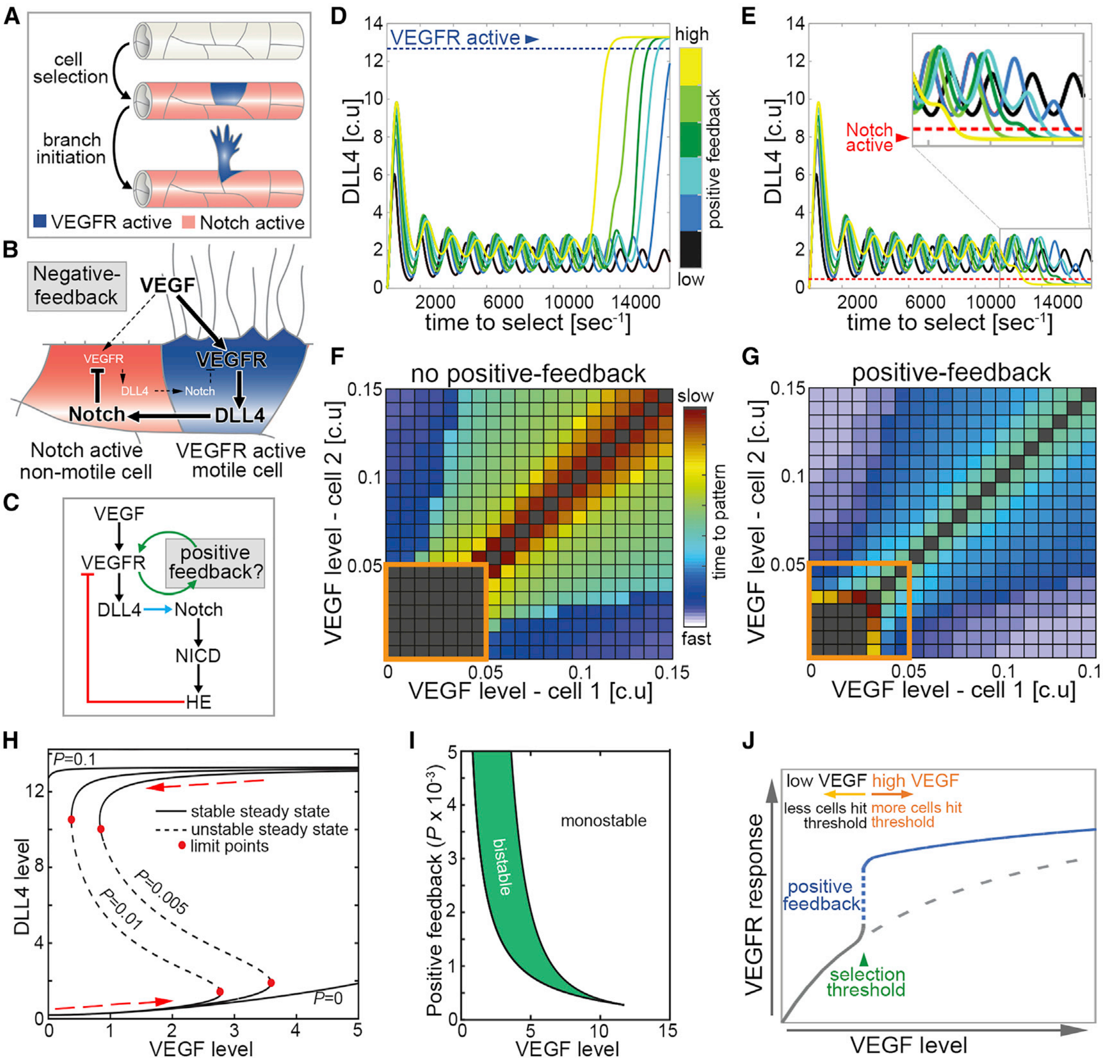

Figure 1. Positive Feedback Generates an Ultrasensitive Angiogenic Switch

(A) In angiogenesis, ECs in quiescent vessels compete for VEGFR-active versus Notch-active status. VEGFR-active cells acquire motile "tip" identity and initiate branching.

(B) An intercellular negative-feedback loop uses lateral inhibition (LI) to limit the number of ECs that acquire VEGFR-active status.

(C) Signaling interactions underpinning construction of the two-cell ODE mathematical model. Blue arrow indicates LI. Green and red arrows indicate positive and negative feedback via VEGFR, respectively. HE refers to the combined effects of Notch-induced expression of transcriptional repressors.

(D and E) Plots of DLL4 levels in two coupled cells following ODE model simulations using varying levels of positive feedback. Depending on their final level of DLL4, each coupled cell was assigned as having acquired either high VEGFR activity and stable tip identity (D; blue arrowhead indicates high levels of DLL4) or high Notch activity and repressed tip identity (E; red arrowhead indicates low levels of DLL4). Blue and red dashed lines represent maximum and minimum DLL4 thresholds for stable tip identity and repressed tip identity, respectively.

(F and G) Matrix plots of tip patterning speeds in the two-cell ODE model following exposure of each coupled cell to different VEGF levels in the absence (F) or presence (G) of positive feedback. Dark gray boxes indicate the slowest rates or failure of tip patterning. Larger orange boxes indicate coupled ECs experiencing low VEGF levels (<0.05 c.u.).

(H) ODE modeling of the impact of positive-feedback levels (*P*) on network bistability. Without positive feedback (*P* = 0), ECs resist switching to a VEGFR active steady state (high DLL4), even when surrounding VEGF is increased. At very high *P* values (*P* = 0.1), ECs remain in a VEGFR active state with changing VEGF. At intermediate *P* values, increasing VEGF levels (>2.5) induce tip cell patterning. Moreover, this active state is retained when VEGF levels are then lowered below 2.5 to ~1. Hence, positive feedback generates a bistable switch in EC identity that robustly maintains the active state, despite fluctuating VEGF levels.

(I) Two-parameter bifurcation plot with changing VEGF and changing *P* values. Region inside the cusp (green shaded portion) represents values that are bistable in the EC active state. Everything outside is monostable.

(J) Predicted role of positive feedback in defining the selection threshold of VEGF that drives tip identity.

Data are mean.

shapes the topology of both normal and pathological vascular networks (Bentley and Chakravartula, 2017; Kur et al., 2016; Ubezio et al., 2016; Venkatraman et al., 2016), our current understanding of the core regulatory features that ultimately spatiotemporally define EC identity is somewhat limited. For example, LI is considered relatively slow, taking upward of 6 h to complete the multiple cycles of gene expression needed to amplify initially small differences in input signal (Bentley and Chakravartula, 2017; Kur et al., 2016; Matsuda et al., 2015; Venkatraman et al., 2016). This is seemingly incompatible with the rapid dynamic changes in EC state, identity, and behavior observed in angiogenesis (Arima et al., 2011; Jakobsson et al., 2010), suggestive of as-yet-unknown temporal modulators that dictate the speed and magnitude of the competitive EC decision-making processes.

Here, by combining computational modeling with *in vivo* studies, we uncover a previously unappreciated role for positive feedback in determining the spatiotemporal dynamics of tip-stalk identity decisions and the angiogenic response. We reveal that Vegfr-mediated expression of the atypical tetraspanin, *transmembrane 4 L six family 18* (*tm4sf18*) generates a previously unknown positive-feedback loop that amplifies Vegfr activity to define the timing, magnitude, and robustness of EC identity decisions. In particular, we propose that positive feedback achieves this by transforming the normally protracted process of LI into a quick, robust switch-like mechanism.

RESULTS

Positive Feedback Creates an Ultrasensitive Bistable Angiogenic Switch

Despite the recognized role of Notch-mediated negative feedback in angiogenesis, the function and identity of positive-feedback modulators remains elusive. First, to explore the impact of positive feedback on the dynamics of EC identity decisions, we adapted our previously validated ordinary differential equation (ODE) mathematical model of LI (Venkatraman et al., 2016), which permits rigorous, mathematical interrogation of the bifurcation dynamics in this system. In this model, two adjacent unpatterned ECs compete for selection as either a VEGFR-active DLL4-expressing tip EC or Notch-active inhibited EC using the well-established VEGFR-DLL4-Notch negative-feedback loop (Figure 1C). Briefly, in this model, VEGF ligand reversibly binds VEGFR to induce DLL4 ligand gene expression, which then reversibly activates the Notch receptor of the neighboring cell. Activated Notch-DLL4 complex is irreversibly catalyzed to form a NICD fragment, which, in turn, induces transcription of the gene repressors. For simplicity, we consolidated all known NICD-induced repressors—namely, the HES, HEY, and HER family proteins—into a single species, HE. Through a negative-feedback mechanism, HE ultimately represses the activity of VEGFR. In addition, we created a parameter, *P*, (see STAR Methods) that creates a positive-feedback interaction whereby VEGFR increases the level and/or activity of an additional factor that positively feeds back to activate more VEGFR. Using previously defined reaction parameters (Bentley et al., 2009, 2014b; Sprinzak et al., 2010; Venkatraman et al., 2016), we revealed that increasing levels of positive feedback could amplify small differences in VEGFR activity between coupled ECs to drive rapid reciprocal VEGFR-Notch activation and patterning of ECs (Figures 1D and 1E). In particular, VEGF-mediated positive feedback was sufficient to transform normally protracted LI into a quick switch-like process and could decrease the level of VEGF required to induce EC identity decisions, as, in the absence of positive feedback, ECs failed to pattern at VEGF levels modeled (Figures 1D and 1E). To explore this phenomenon further, we investigated the impact of varying the levels of VEGF initially experienced by each cell in the ODE model. The times taken for coupled cells to stably pattern in the absence or presence of positive feedback were then represented as time matrix plots (Figures 1F and 1G). As described earlier, positive feedback greatly increased the speed of LI-mediated patterning, particularly when coupled ECs experienced similar levels of VEGF that normally makes it difficult to discern differences in their VEGFR activity. Importantly, positive feedback notably decreased the threshold of VEGF required to induce stable patterning, as ECs could make definitive identity decisions at much lower levels of VEGF than possible in the absence of positive feedback (orange boxes in Figures 1F and 1G). Hence, *in silico* modeling predicted that positive feedback defines the threshold of VEGF required to induce motile EC selection and greatly increases the speed of EC decision making by invoking ultrasensitive switch-like behavior during LI.

As well as creating ultrasensitive signaling switches, a core feature of positive feedback is that it contributes to the establishment of bistable networks, which, in turn, can confer robustness on cell-state transitions by using hysteresis (Brandman and Meyer, 2008; Freeman, 2000). In hysteresis, the state in which a system resides depends not only on the current conditions but also on the history of the system. As such, in cellular systems, hysteresis enables the same level of input signal to have two very distinct cellular outputs, depending on the system's history. For example, rising levels of an input signal may elicit highly stereotyped cellular outputs, but in hysteresis, the system will not follow these same steps in reverse when returning to back to the original level of signal. Hence, hysteresis can induce stable switch-like behavior if, as a consequence of achieving a sufficient signal to drive cell-state transition, much lower levels of this signal are now required to reverse that cell state. Thus, hysteresis can reinforce robust cell identity decisions by ensuring that, once cell identity is determined, fluctuating levels of signal will not reverse that decision. Further extension of the ODE modeling revealed that intermediate levels of VEGFR-mediated positive feedback generated typical hysteretic dynamics during LI *in silico* (Figure 1H). At specific levels of positive feedback, LI-mediated EC identity decisions were, indeed, bistable (Figure 1I) and, once made, were highly robust to subsequent decreases in VEGF level, indicating hysteresis (Figure 1H). Hence, as well as invoking switch-like behavior during EC decision making, positive feedback may also confer robustness on selected EC identity against fluctuations in inductive VEGF signal.

Switch-like Control of Angiogenesis *In Vivo* by the Vegfr-Notch Axis

Simulations predicted that positive feedback invokes switch-like dynamics during LI whereby, if a threshold of VEGF is achieved,

OPEN ACCESS
CellPress

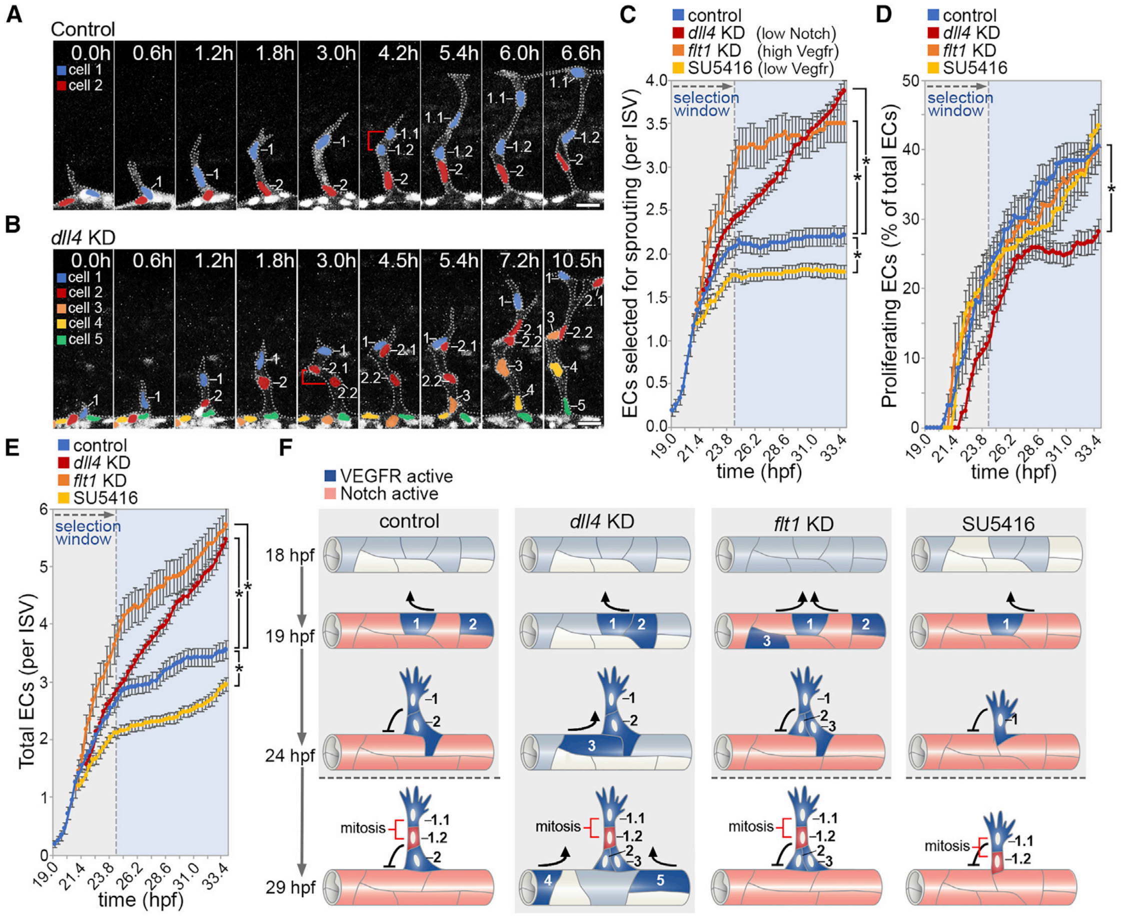

Figure 2. Switch-like Behavior of Motile EC Selection in Angiogenesis *In Vivo*

(A and B) Time-lapse images of EC nuclei in ISVs of control (A) and *dll4* KD (B) *Tg(kdrl:nlsEGFP)*^{zf109} embryos from 19 h post-fertilization (hpf). Brackets indicate dividing cells. Nuclei are pseudocolored.

(C–E) Quantification of the number of ECs that are selected to branch (C), undergo proliferation (D), or the total number of ECs per ISV (E) in control, *dll4* KD, *flt1* KD, and 0.3 μM SU5416-treated embryos (n = 47 ISVs from 16 control, 78 ISVs from 24 *dll4* KD, 28 ISVs from 8 *flt1* KD, and 81 ISVs from 23 0.3 μM SU5416-treated embryos).

(F) Illustration of the biphasic nature of the selection of motile ECs in angiogenesis. Vegf signal levels define the number of ECs selected to branch, and Dll4-mediated LI prevents further selection of motile ECs. Increased Vegf (*flt1* KD) or decreased Vegf (0.3 μM SU5416) signaling results in the selection of more or less motile ECs, respectively. In the absence of *dll4*, motile ECs continue to be selected.

Data are mean ± SEM. *$p < 0.05$, two-way ANOVA test. Scale bars, 25 μm.

See also Figure S1.

positive-feedback-mediated amplification of signal ensures rapid commitment of ECs for patterning and selection (Figures 1D–1I). As such, VEGF levels may ultimately dictate the magnitude of an angiogenic response *in vivo* by determining how many ECs achieve a selection threshold and are triggered to pattern (Figure 1J). However, we currently have little to no knowledge of the magnitude or timing of such EC decision-making processes *in vivo*. Hence, we first probed the dynamics of LI-mediated motile EC selection during zebrafish intersegmental vessel (ISV) angiogenesis. Live imaging of ISV sprouting in *Tg(kdrl:nlsEGFP)*^{zf109} zebrafish embryos revealed that LI defined a tight temporally restricted selection window that robustly generated, on average, just two motile ECs per vessel by 24 h post-fertilization (hpf; Figures 2A and 2C). As was expected, Notch signaling was required to close this window, as in the absence of *dll4* or upon blockade of Notch signaling using γ-secretase inhibitors, the rate of EC selection did not initially change but continued unstopped (Figures 2B and 2C; Figure S1A). Importantly, consistent with *in silico* predictions (Figure 1J), Vegfr levels determined the number of ECs selected within this window, as loss of *flt1* to enhance Vegfr signaling increased the number of ECs selected to sprout (Figure 2C; Figure S1B). Likewise, the opposite was observed upon brief low-dose inhibition of Vegfr signaling, with fewer cells selected (Figure 2C; Figure S1C). As we cannot interrogate signaling dynamics *in vivo*, we utilized the well-validated Memagent-Spring (MSM) model of Vegf-Notch selection to simulate how cells collectively compete within the DA prior to sprouting (Bentley et al., 2014a;

Costa et al., 2016; Villefranc et al., 2013) (STAR Methods). Using exactly the model parameters as previously published, a single early time window could, indeed, be found that remarkably exhibited exactly matching phenomena as seen *in vivo* (Figures S1D–S1F), whereby fewer ECs were selected in Vegf-inhibited conditions (VEGF = 0.038), and more were selected in *flt1* knockdown (KD) conditions (V_{sink} = 8; see STAR Methods for a full description of these model parameters). Moreover, monitoring of signaling dynamics in simulations confirmed that Vegf levels determined the number of selected ECs by modulating the speed of the selection process, as also predicted in the ODE model (Figure 1).

Importantly, differences in Vegfr-dependent selection rates *in vivo* were not associated with differential EC proliferation (Figure 2D), although a later switch to Notch-dependent mitosis was revealed by *dll4* KD. Moreover, the contribution of ECs to ISVs by proliferation after 24 hpf had minimal effect on overall EC numbers, with both the initial magnitude of EC selection and the Notch-dependent closure of the selection window being the primary determinants of vessel cellularity (Figure 2E). Hence, by defining the temporal control of angiogenesis *in vivo*, we revealed that EC identity decisions are biphasic, involving (1) a Vegfr-level-dependent switch that determines the number of motile ECs selected for angiogenesis, followed by (2) termination of further selection by Dll4-Notch (Figure 2F).

Vegfr-Induced Expression of *tm4sf18* Generates Positive Feedback *In Vivo*

Both *in silico* predictions (Figure 1) and the switch-like nature of the EC selection (Figure 2C) suggested that motile EC selection in angiogenesis may, indeed, be modulated by a positive-feedback-mediated ultrasensitive switch. To support these observations, we expanded upon our previous transcriptomic study (Herbert et al., 2012) to identify putative positive-feedback regulators of Vegfr by defining genes transcriptionally activated by Vegfr and repressed by Notch signaling in zebrafish ECs (Figure 3A). Of only 10 candidate Vegfr-Notch-regulated transcripts we identified *h2.0-like homeobox-1* (*hlx1*), a known transcriptional target of Vegfr activity *in vivo* (Fish et al., 2017; Herbert et al., 2012; Sacilotto et al., 2016) and the atypical tetraspanin, *tm4sf18*. TM4SF family proteins are known membrane-associated adaptors that ligand-independently activate receptor tyrosine kinase activity (Gao et al., 2016; Jung et al., 2013). Hence, of the 10 identified Vegfr-regulated transcripts, *tm4sf18* was the only one with clear evidence supporting a role in the feedback control of Vegfr receptor tyrosine kinase activity. Indeed, *TM4SF1*, the human homolog of *tm4sf18* (Figure S2), is known to modulate EC motile behavior *in vitro* (Shih et al., 2009; Zukauskas et al., 2011). Hence, for these reasons and other technical points (STAR Methods), *tm4sf18* represented an ideal candidate positive-feedback modulator of EC motile identity for further investigation. The highly dynamic nature of Vegfr-Notch-regulated expression of *tm4sf18* was validated via qPCR upon the inhibition of Vegfr signaling and *dll4* KD (Figures 3B and 3C), indicating that *tm4sf18* transcription may be tightly restricted to sprouting EC populations by Vegf-Notch (Figure 3D). This was further confirmed following characterization of the spatiotemporal pattern of *tm4sf18* expression during zebrafish development. During early ISV sprouting from 22 to 26 hpf, *tm4sf18* expression was almost exclusively restricted to sprouting ISVs (blue brackets in Figure 3E) and was excluded from adjacent non-angiogenic vascular tissues, such as the dorsal aorta (DA). Indeed, consistent with a role for Tm4sf18 in the amplification of Vegfr activity and EC selection, expression of *tm4sf18* was also observed prior to ISV sprouting at discrete foci marking regions of future angiogenic remodeling within the DA (arrowheads in Figure 3E). Importantly, the absence of *tm4sf18* in *npas4l*^{s5} mutants that lack endothelial tissues (Reischauer et al., 2016) confirmed expression in ECs (Figure 3F). Moreover, *tm4sf18* expression was ectopically expanded to non-angiogenic tissues upon *dll4* KD, demonstrating a tight association with EC sprouting (Figure 3F). Indeed, rapid repression of *tm4sf18* was observed following fusion of adjacent ISVs to form the dorsolateral anastomotic vessel (DLAV) and termination of Vegf-induced angiogenic behavior (red brackets in Figure 3E). Hence, *tm4sf18* expression is dynamically modulated by the Vegfr-Notch axis and is spatiotemporally restricted to sprouting ECs *in vivo*.

To define *tm4sf18* as a putative positive-feedback modulator of VEGF signaling, we transfected human umbilical vein ECs (HUVECs) with small interfering RNAs (siRNAs) targeting the human homolog of zebrafish *tm4sf18*, *TM4SF1*, which reduced mRNA abundance by over 80% (Figure 4A). Subsequent stimulation of HUVECs with VEGF-A confirmed that maximal VEGFR-dependent ERK activation relied on *TM4SF1* expression (Figures 4B and 4C), consistent with the known role for TM4SF family proteins in the activation of receptor tyrosine kinase signaling (Gao et al., 2016; Jung et al., 2013). To confirm these observations *in vivo*, we used both TALEN and CRISPR/Cas9-mediated gene editing to introduce mutations into the long (exons 1 to 4) or both long and short (exons 2 to 4) isoforms of *tm4sf18*, respectively (Figure 4D). TALEN-mediated nonsense mutation of *tm4sf18* introduced a premature stop codon in exon 1 at amino acid 17, whereas CRISPR/Cas9-mediated gene editing introduced a frameshift mutation from amino acid 68 in exon 2 and generated a truncated protein product of 112 amino acids versus wild-type (WT) Tm4sf18 (196 amino acids). Importantly, upon *tm4sf18* exon-1 and exon-2 mutation, we observed no change in tip EC levels of Vegfr-dependent EC pErk (data not shown), a well-established readout for Vegfr signaling *in vivo* (Costa et al., 2016; Nagasawa-Masuda and Terai, 2016; Shin et al., 2016). Thus, in the absence of Tm4sf18, at least some ECs still achieve Vegfr activity thresholds sufficient to drive tip patterning. However, the temporal dynamics of Vegfr signaling were significantly perturbed in *tm4sf18*$^{-/-}$ homozygous mutant embryos (Figures 4E–4I). To test this, first, Vegfr activity was fully blocked in the ECs of sprouting ISVs that had already emerged from the DA upon incubation of embryos for 3 h with the Vegfr inhibitor, ZM323881 (Figure 4E). Full disruption of Vegfr activity was confirmed by a lack of immunoreactivity for pErk (Figures 4F and 4G). Moreover, disruption of pErk was specific to ECs, as levels remained unchanged in neighboring neuronal cells (Figures 4F and 4H). Inhibitor was then washed out, and the recovery of EC pErk levels was monitored over the next 4 h. Quantification of pErk in sprouting tip ECs revealed that normal levels were recovered after just 2 h in both control and *tm4sf18*$^{+/-}$ heterozygous embryos (Figures 4F and 4G). However, recovery of

OPEN ACCESS
CellPress

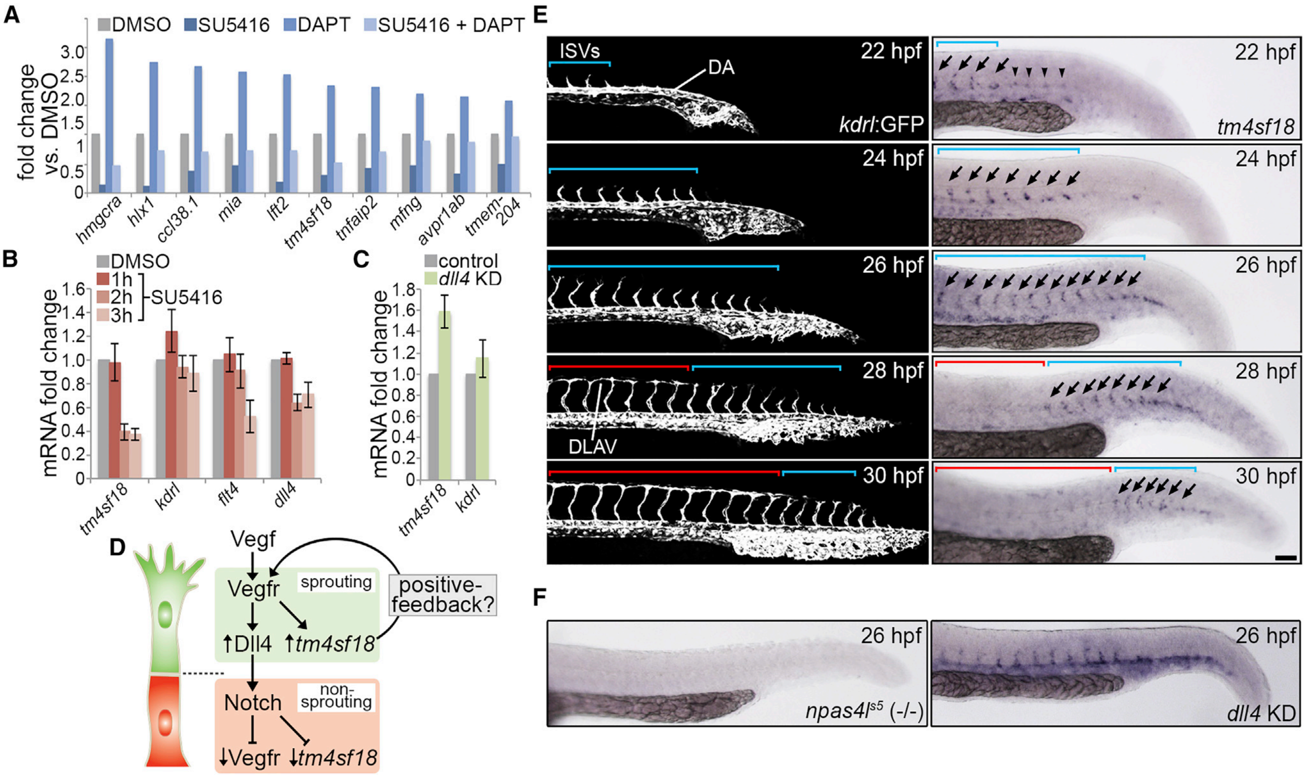

Figure 3. Identification of Putative Positive-Feedback Modulators of Vegf Signaling

(A) Fold change in the indicated transcript levels by microarray following inhibition of Vegfr signaling (2.5 μM SU5416), Notch activity (100 μM DAPT), or both, from 22 to 30 hpf.

(B and C) Fold change in *tm4sf18*, *kdrl*, *flt4*, and *dll4* transcript levels by qPCR in embryos incubated with 2.5 μM SU5416 for the indicated times (B) and *tm4sf18* and *kdrl* transcript levels by qPCR upon *dll4* KD (C; n = 3 separate experiments).

(D) Illustration of the putative transcriptional regulation of *tm4sf18* by Vegf-Notch and proposed function as a positive-feedback modulator of Vegfr signaling.

(E) Lateral views of sprouting ISVs in *Tg(kdrl:GFP)*^{s843} embryos (left) or WT embryos following whole-mount *in situ* hybridization analysis of *tm4sf18* expression (right). Blue brackets indicate nascent ISVs; red brackets indicate anastomosed ISVs; arrows indicate *tm4sf18*-expressing ISVs; and arrowheads indicate *tm4sf18* expression at regions of future angiogenic remodeling.

(F) Whole-mount *in situ* hybridization analysis of *tm4sf18* expression in *npas4l*^{s5} mutant embryos showing loss of expression, as well as upon *dll4* KD showing ectopic expansion of *tm4sf18* expression to the DA, consistent with de-repression of Vegfr signaling.

Data are mean ± SEM. Scale bar, 100 μm.

See also Figure S2.

Vegfr-mediated pErk stalled after 1 h of recovery and was significantly disrupted in *tm4sf18*$^{-/-}$ mutants (Figures 4H and 4I). Hence, consistent with a key functional role as a positive-feedback modulator of Vegf, expression of *tm4sf18/TM4SF1* amplifies EC signaling both *in vivo* and *in vitro* and facilitates the acquisition of high-level Vegfr activity.

Tm4sf18 Determines the Magnitude and Timing of EC Identity Decisions *In Vivo*

To define the potential functional role of Tm4sf18-mediated positive feedback in EC decision making *in vivo*, we quantified the rate of EC selection during ISV sprouting to reveal a significant reduction in *tm4sf18*$^{-/-}$ embryos (Figure 5A). Observed defects in EC selection were specific to ISV sprouting, as later branching of the venous secondary sprouts was unaffected in *tm4sf18* mutants (Figure S3A). In addition, reduced selection of motile ECs was not simply a consequence of developmental delay or decreased EC proliferation, as developmental timing, proliferative rates, and cell-cycle length were all unperturbed by *tm4sf18* mutation (Figure 5B; Figures S3B and S3C). Moreover, in the absence of Tm4sf18-dependent selection of motile ECs, nascent ISVs exhibited a persistent hypocellular phenotype (Figure 5C and 5D), although this recovered at later time points, presumably due to compensation by sustained EC proliferation and later supply of ECs via secondary sprouting (Figure S3D). These findings were consistent with model predictions that positive-feedback-mediated amplification of Vegf signal may lower the threshold of Vegf required to induce patterning. Hence, in the absence of positive feedback, the levels of Vegf required to induce patterning would be higher, and fewer ECs would be able to achieve these within the temporally defined selection window (Figure 5E). Indeed, consistent with this hypothesis, the disruption of motile EC selection in *tm4sf18* mutants could largely be recovered when Vegf signal levels were increased

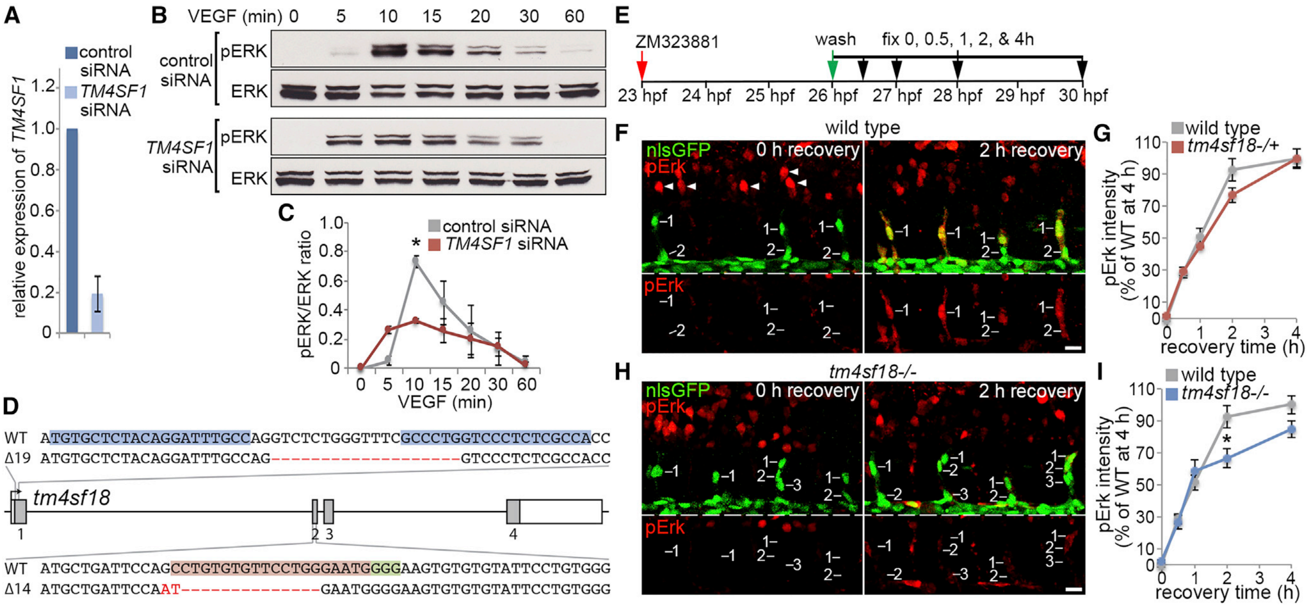

Figure 4. TM4SF1/Tm4sf18 Expression Feeds Back to Amplify VEGF/Vegf Signaling

(A) Relative expression levels of *TM4SF1* by qPCR in HUVECs transfected with control or TM4SF1-targeted siRNA (n = 4 separate experiments).

(B and C) Western blot analysis of pERK/ERK levels in HUVECs after VEGF-A stimulation following transfection with control or *TM4SF1*-targetting siRNA (B) and quantification of pERK/ERK ratios (C) (n = 3 separate experiments).

(D) Lesions introduced into the *tm4sf18* loci by TALEN and CRISPR gene editing. A 19-bp deletion of *tm4sf18* exon-1 and a 16-bp deletion and 2-bp insertion of exon-2 were generated using TALENs and CRISPR/Cas9, respectively. Genomic target sites for the TALENs, gRNA target site, and PAM sequence are indicated by blue, red, and green highlights, respectively.

(E) Strategy for assessing Vegfr signaling dynamics *in vivo*.

(F–I) Lateral views of pErk immunostaining in ECs of WT (F) or $tm4sf18^{-/-}$ (H) $Tg(kdrl:nlsEGFP)^{zf109}$ embryos at 0 and 2 h after inhibitor washout and quantification of pErk fluorescence intensity in WT, $tm4sf18^{+/-}$ (G) or $tm4sf18^{-/-}$ (I) embryos. Arrowheads in (F) indicate pErk in neuronal cells (n = at least 39 ECs from 8 WT, 129 ECs from 20 $tm4sf18^{+/-}$, and 74 ECs from 13 $tm4sf18^{-/-}$ embryos at each time point).

Data are mean ± SEM. *p < 0.05, two-tailed t test. Scale bars, 25 μm.

via *flt1* KD (Figure S3E). To further test this hypothesis, we reasoned that additional inhibition of Vegfr activity in $tm4sf18^{-/-}$ mutants would now prevent all ECs from quickly achieving a selection threshold, potentially delaying the timing of EC selection. As such, we blocked EC Vegfr activity with a low dose of Vegfr inhibitor to putatively force ECs to be reliant on positive-feedback amplification of Vegfr activity. Indeed, upon Vegfr inhibition, not only was the emergence of the first selected ECs now greatly delayed in $tm4sf18^{-/-}$ mutants (Figure 5F and 5G), but this treatment also generated a large delay to the EC selection window (Figure 5F), independent of any effects on EC proliferation (Figure S3F). Hence, not only does Tm4sf18 control the magnitude of EC sprouting, when Vegfr activity is limiting, but Tm4sf18-mediated positive feedback also ensures timely decision making.

Tm4sf18-Modulated Vessel Cellularity Is Critical for Normal Angiogenesis

In parallel with a reduced number of ECs selected for branching, we noted that resulting hypocellular ISVs in $tm4sf18^{-/-}$ embryos were often shorter (Figure 6A), an observation that was confirmed upon quantification of EC tip (cell 1) and stalk (cell 2) movement in WT, $tm4sf18^{+/-}$, and $tm4sf18^{-/-}$ embryos (Figures 6B and 6C). This phenotype was reliant on mutation of both short and long Tm4sf18 isoforms, as exon-1 mutant embryos were unaffected (Figure S4A), and was not due to indirect differences in ISV morphology (Figure S4B). Importantly, although *tm4sf18*-mediated EC emergence and motility appeared to be VEGF dependent (Figure S4C), disruption of ISV extension was not a consequence of reduced EC motility, as movement of emerging $tm4sf18^{-/-}$ ECs was initially indistinguishable from that of WT, and stalling was only observed later in development (Figure 6C). Strikingly, however, we observed a near-identical phenotype upon disruption of EC proliferation using hydroxyurea and aphidicolin (HU/Ap; Figure 6D), suggesting common underlying defects. Although HU/Ap treatment did not disrupt EC motile selection, unlike loss of *tm4sf18* (Figure 5A versus Figure S3G), and mutation of *tm4sf18* did not disrupt EC proliferation, unlike HU/Ap (Figure 5B versus S3H), both *tm4sf18* mutation and HU/Ap treatment generated very similar and prolonged reductions in ISV cellularity (Figures 5D and S3I). Moreover, quantification of tip EC motility in ISVs containing 1, 2, and 3 or more ECs revealed that it is this level of vessel cellularity that determines vessel extension (Figure 6E). Indeed, the perturbed extension of vessels observed in ECs lacking Tm4sf18 was consistent with the reduced average number of ECs per ISV in $tm4sf18^{-/-}$ mutants of approximately two per vessel (Figure 5C). Likewise, WT and HU/Ap-treated tip ECs display movements consistent

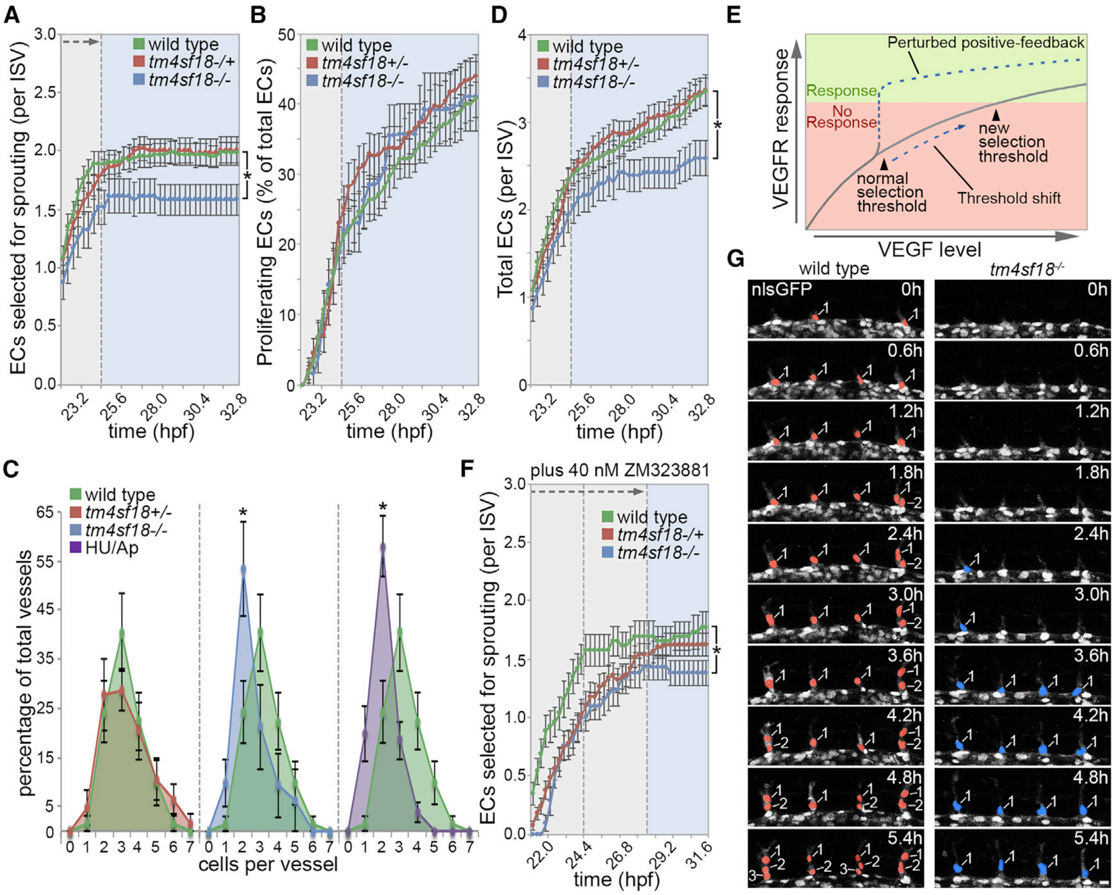

Figure 5. Tm4sf18 Modulates the Magnitude and Timing of the Angiogenic Response

(A and B) Quantification of the number of ECs selected to branch (A) or the percentage of ECs that undergo proliferation (B) in WT, *tm4sf18*$^{+/-}$, and *tm4sf18*$^{-/-}$ embryos (n = 62 ISVs from 16 WT, 58 ISVs from 15 *tm4sf18*$^{+/-}$, and 31 ISVs from 8 *tm4sf18*$^{-/-}$ embryos).

(C) Quantification of the distribution of ISV cellularity in WT, *tm4sf18*$^{+/-}$, *tm4sf18*$^{-/-}$, and HU/Ap-treated embryos (n = 65 ISVs from 16 WT, 62 ISVs from 15 *tm4sf18*$^{+/-}$, 31 ISVs from 8 *tm4sf18*$^{-/-}$, and 88 ISVs from 22 HU/Ap-treated embryos).

(D) Quantification of the total number of ECs per ISV in WT, *tm4sf18*$^{+/-}$, and *tm4sf18*$^{-/-}$ embryos. n is the same as in (A).

(E) Predicted shift in the level of VEGF signaling required to achieve a selection threshold in the absence of positive feedback.

(F and G) Quantification of the number of ECs selected to branch in 40 nM ZM323881-treated WT, *tm4sf18*$^{+/-}$ and *tm4sf18*$^{-/-}$ embryos (F) and corresponding time-lapse images of sprouting ISVs in 40 nM ZM323881-treated WT and *tm4sf18*$^{-/-}$ embryos from 20 hpf (G). Embryos were incubated with 40 nM ZM323881 from 18 hpf onward. Nuclei of sprouting ECs emerging from the DA are pseudocolored (n = 26 ISVs from 10 WT, 50 ISVs from 20 *tm4sf18*$^{+/-}$, and 21 ISVs from 10 *tm4sf18*$^{-/-}$ embryos).

Data are means ± SEM. *p < 0.05, two-way ANOVA or two-tailed t test. Scale bar, 25 μm.

See also Figure S3.

with the average cellularity of vessels in these conditions (Figure S4D). Consequently, both *tm4sf18*$^{-/-}$ mutation and HU/Ap treatment significantly perturb the supply of ECs to form the DLAV (Figure 6F). Hence, Tm4sf18-mediated positive feedback determines the correct number of ECs selected for vessel branching, and, in its absence, nascent hypocellular vessels fail to extend appropriately (Figure 6G).

Tm4sf18-Mediated Positive Feedback Promotes Robust EC Decision Making

A functional role for Tm4sf18 in determining the magnitude and timing of EC identity specification are consistent with model predictions that positive feedback generates an ultrasensitive switch that modulates the decision-making process (Figure 1). However, a core feature of such ultrasensitive switches is that they can also invoke bistability and hysteretic dynamics, which could confer robustness to selected EC identity against variation in inductive VEGF signal levels (Brandman and Meyer, 2008; Freeman, 2000). To test these predictions, we waited until after ECs were selected for branching, i.e., already in ISV sprouts (>22 hpf) and, hence, already above selection thresholds of Vegfr activity. Then we determined the robustness of tip EC signaling to increasing concentrations of Vegfr inhibitor for 3 h. In WT and *tm4sf18*$^{+/-}$ embryos, Vegfr signaling was highly robust to Vegfr antagonist, with no significant reduction in pErk levels observed upon incubation with 40 nM and 80 nM ZM323881 (Figures 7A

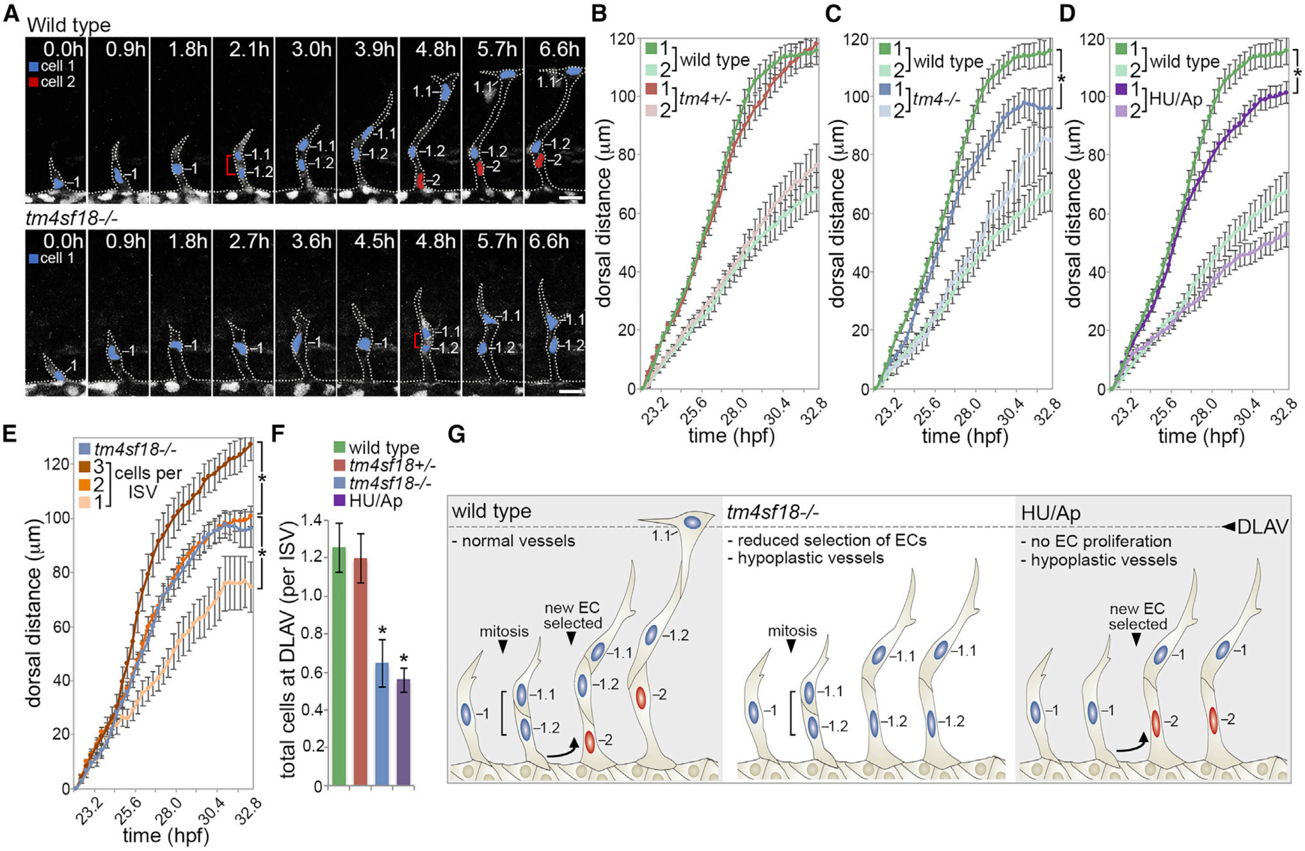

Figure 6. Hypocellular Vessels in *tm4sf18*$^{-/-}$ Mutants Fail to Extend Appropriately

(A) Time-lapse images of sprouting ISVs in WT and *tm4sf18*$^{-/-}$ *Tg(kdrl:nlsEGFP)*^{zf109} embryos from 19 hpf. Brackets indicate dividing cells. Nuclei are pseudocolored. ISVs appear shorter in the absence of *tm4sf18*.

(B–D) Quantification of the dorsal movement of tip (cell 1) or stalk (cell 2) ECs in WT and *tm4sf18*$^{+/-}$ (B), *tm4sf18*$^{-/-}$ (C), or HU/Ap-treated (D) embryos (n = 71 ISVs from 16 WT, 69 ISVs from 15 *tm4sf18*$^{+/-}$, 39 ISVs from 8 *tm4sf18*$^{-/-}$, and 89 ISVs from 22 HU/Ap-treated embryos).

(E) Quantification of the dorsal movement of tip ECs in non-proliferating ISVs consisting of 1, 2, and 3 or more ECs and comparison with the motility of tip ECs in *tm4sf18*$^{-/-}$ embryos (n = 39 ISVs from 8 *tm4sf18*$^{-/-}$ embryos, as well as 17 ISVs with 3 cells, 53 ISVs with 2 cells, and 16 ISVs with 1 cell from 22 embryos).

(F) Quantification of the number of ECs that reach the DLAV position in WT, *tm4sf18*$^{+/-}$, *tm4sf18*$^{-/-}$, and HU/Ap-treated embryos (n = 63 ISVs from 16 WT, 56 ISVs from 15 *tm4sf18*$^{+/-}$, 31 ISVs from 8 *tm4sf18*$^{-/-}$, and 86 ISVs from 22 HU/Ap-treated embryos).

(G) Illustration of the causes of vessel hypoplasia and phenotypic effect on vessel extension.

Data are means ± SEM. *$p < 0.05$, two-way ANOVA or two-tailed t test. Scale bars, 25 μm.

See also Figure S4.

and 7B). In contrast, Vegfr signaling was no longer protected in *tm4sf18*$^{-/-}$ embryos, and pErk levels were significantly disrupted upon incubation of embryos with a low dose of inhibitor. Hence, consistent with *in silico* predictions, Tm4sf18-mediated positive feedback generates signal robustness reminiscent of a bistable network and ultimately buffers Vegfr signaling output against fluctuations in input signal to generate robust angiogenic responses (Figure 7C).

DISCUSSION

Using an integrated *in silico* and *in vivo* approach, we provide evidence that positive feedback is a key spatiotemporal modulator of angiogenesis. Specifically, we demonstrate that positive feedback amplifies Vegfr-mediated signaling to generate a previously unappreciated ultrasensitive switch that (1) defines the timing of competitive EC identity decisions, (2) controls the magnitude of angiogenic responses, and (3) elegantly confers developmental robustness on angiogenesis against fluctuations in pro-angiogenic signal (Figure 7D). Moreover, we define Tm4sf18 as a previously unknown positive-feedback modulator of Vegfr activity that performs these functions *in vivo*. We reveal that Vegfr signaling dynamically drives expression of *tm4sf18* in pre-angiogenic ECs, which feeds back to amplify Vegfr signaling and promote rapid selection of motile ECs during angiogenesis. Importantly, we propose that Tm4sf18-mediated positive feedback may ultimately achieve this by magnifying subtle differences in Vegf signal levels between competing ECs to significantly expedite LI-mediated EC identity decisions. Hence, we present evidence that positive feedback can transform the normally protracted mechanism of LI into a rapid, switch-like decision-making process. As a consequence, in the absence

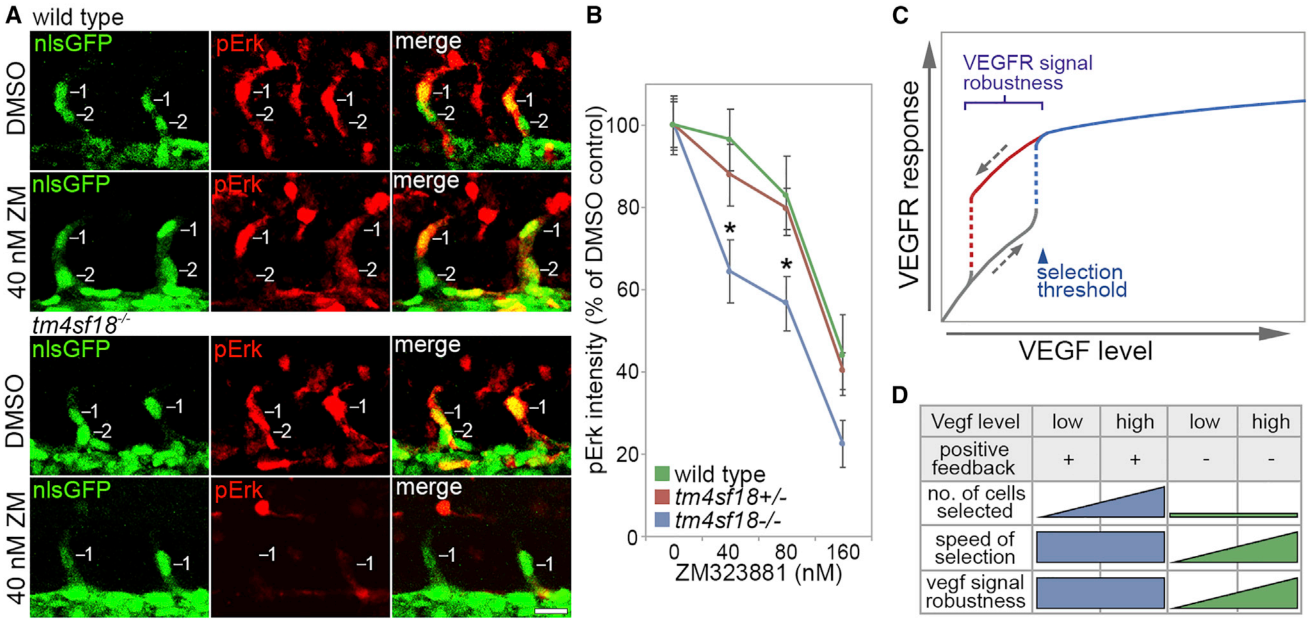

Figure 7. Robustness of Tip Identity Is Lost in *tm4sf18*$^{-/-}$ Mutants

(A) Lateral views of sprouting ECs in ISVs of WT and *tm4sf18*$^{-/-}$ *Tg(kdrl:nlsEGFP)*^{zf109} embryos immunostained for pErk. Prior to fixation, embryos were incubated with DMSO or 40 nM ZM323881 from 22 hpf for 3 h.

(B) Quantification of pErk fluorescence intensity in WT, *tm4sf18*$^{+/-}$, and *tm4sf18*$^{-/-}$ embryos following incubation with DMSO or increasing concentrations of ZM323881 (n = at least 32 ECs from 8 WT, 87 ECs from 22 *tm4sf18*$^{+/-}$, and 35 cells from 8 *tm4sf18*$^{-/-}$ embryos at each concentration).

(C) Putative role of positive-feedback-generated bistability and hysteretic dynamics in the control of VEGFR signal level robustness in angiogenesis. Bistability ensures that higher levels of VEGF are required to induce tip patterning than to reverse this active state, conferring robustness on tip identity against fluctuations in VEGF levels.

(D) Impact of Tm4sf18-mediated positive feedback on the magnitude, speed, and robustness of motile EC selection during ISV branching. Tm4sf18 drives quick and robust decision making but also ensures delicate modulation of the magnitude of EC selection by Vegf levels. In the absence of Tm4sf18, the magnitude of EC selection is diminished, and both the speed and robustness of EC selection are highly variable and more dependent on Vegf level.

Data are means ± SEM. *$p < 0.05$, two-way ANOVA or two-tailed t test. Scale bar, 25 μm.

of Tm4sf18, the speed of motile EC selection becomes highly sensitive to fluctuations in Vegf signaling and is notably delayed even by partial inhibition of Vegfr activity. Recent work demonstrates that such temporal control of EC LI fundamentally defines the topology of newly forming vascular networks, with faster rates of tip EC selection dramatically increasing vessel network density (Kur et al., 2016). Consistent with this work, we find that the slower rates of EC selection linked to loss of Tm4sf18-mediated positive feedback result in a significant reduction in both the number of ECs selected to branch in angiogenesis and the cellularity of nascent vessels. As a consequence, these hypocellular vessels fail to appropriately extend, suggesting that intrinsic adjustment of positive-feedback levels could represent an adaptable framework for the context-dependent modulation of vascular network cellularity and/or topology *in vivo*. Indeed, Tm4sf18-mediated positive feedback appears to be specific to Vegf-a-driven arterial EC sprouting, as Vegf-c-mediated venous secondary sprouting is unaffected by *tm4sf18* mutation. As such, it will be important to determine whether Tm4sf18-mediated control of Vegfr activity and LI are, indeed, differentially regulated in distinct vessel networks and, if that is the case, whether this is indicative of broader functional roles for positive feedback in differentially shaping the topology and/or branch density of specialized vascular beds.

We also reveal that positive feedback uniquely confers developmental robustness on angiogenesis against fluctuations in Vegfr activity. Using computational modeling, we predicted that positive-feedback-mediated amplification of Vegfr signaling efficiently lowers the threshold of Vegfr activity required to promote robust tip cell selection. Consequently, positive feedback transforms the process of LI into an ultrasensitive bistable switch and invokes hysteretic dynamics that ultimately stabilize selected EC identities. We further show that selection of motile ECs exhibits such bistable-like dynamics *in vivo* and, indeed, that Tm4sf18-mediated positive feedback maintains robust tip identity by ensuring that high Vegfr signaling outputs are achieved even when Vegfr activation is perturbed. Consequently, even subtle perturbation of Vegfr activity severely disrupts tip EC Vegfr activity in *tm4sf18* mutant embryos, whereas WT clutchmates remain unperturbed. In particular, these observations hint that human *TM4SF* genes could potentially be exploited in therapeutic contexts. For example, abnormally high levels of VEGFR signaling were recently shown to drive synchronous oscillations of Dll4 in neighboring EC, underpinning a switch from normal EC communal branching behavior to the pathological vessel expansion associated with human retinopathies (Ubezio et al., 2016). Disruption of positive feedback would significantly increase the threshold of VEGFR activity

required to switch vessels to abnormal expansion, putatively blocking progression to synchronous oscillatory dynamics and defining a potential therapeutic approach to normalize branching. Likewise, disruption of positive feedback could increase the potency of existing VEGFR-targeting anti-angiogenic anti-cancer therapeutics by reducing the concentration of compound required to block functional angiogenesis. Moreover, our work opens up the exciting possibility that other known modulators of VEGFR signaling may perform complementary positive-feedback functions to fine-tune the timing and robustness of the VEGF pathway. For example, the secreted protein EC-specific molecule-1 (Esm1) is known to activate VEGFR by increasing the local bioavailability of VEGF (Rocha et al., 2014). Hence, VEGF-induced expression of *Esm1* in sprouting ECs could also confer robustness to VEGF signaling, much like *tm4sf18*, by forming a complimentary non-cell-autonomous positive-feedback loop with VEGFR. Indeed, it is even possible that the other Vegfr-regulated transcripts identified in this study play complementary positive-feedback roles. As such, it will be important to revisit this previous work to fully understand the complexities of feedback control of EC LI.

Overall, our observations reveal that the relatively slow dynamics of LI-mediated cell-fate decisions can be transformed into quick, adaptive, and robust decision-making processes by simply incorporating positive feedback. Considering that LI underpins many cell-fate decisions driving tissue formation, homeostasis, and repair, it is tempting to speculate that such spatiotemporal adaptation by positive feedback may shape collective cell-fate decisions in diverse tissue contexts.

STAR★METHODS

Detailed methods are provided in the online version of this paper and include the following:

- KEY RESOURCES TABLE
- CONTACT FOR REAGENT AND RESOURCE SHARING
- EXPERIMENTAL MODEL AND SUBJECT DETAILS
  - Animals
  - Primary cell culture
- METHOD DETAILS
  - Time-lapse imaging
  - Morpholino oligonucleotide (MO) injections
  - Pharmacological treatments
  - Simulations with the memAgent-spring model (MSM)
  - ODE model construction & simulation
  - Isolation of zebrafish ECs and transcriptome analyses
  - Quantitative real-time PCR (qPCR)
  - Cloning of tm4sf18 and whole-mount *in situ* hybridization
  - siRNA-mediated gene knockdown
  - Immunoblotting
  - Phylogenetic analysis
  - Gene editing
  - pErk immunostaining
- QUANTIFICATION AND STATISTICAL ANALYSIS
- DATA AND SOFTWARE AVAILABLITY

SUPPLEMENTAL INFORMATION

Supplemental Information can be found online at https://doi.org/10.1016/j.celrep.2019.05.052.

ACKNOWLEDGMENTS

We thank B. Plusa, R. Das, K. Dorey, and members of the Herbert lab for helpful discussion and suggestions. We thank members of the University of Manchester Biological Services Facility for providing excellent fish husbandry. S.P.H. was funded by the Wellcome Trust (Ref. 095718/Z/11/Z), BBSRC (Ref. BB/N013174/1), and BHF (PG/16/2/31863). K.B. was funded by BIDMC and NSF (Ref. 1517390) and by the Knut and Alice Wallenberg Foundation. T.T. was funded by the Leverhulme Trust (Ref. RPG-2014-370).

AUTHOR CONTRIBUTIONS

Conceptualization, S.P.H. and K.B.; Methodology, S.P.H., K.B., D.J.P., R.T., and L.V.; Software, K.B. and L.V.; Investigation, S.P.H., D.J.P., R.T., L.V., and T.T.; Resources, K.B., L.V., and T.T.; Writing – Original Draft, S.P.H. and K.B.; Writing – Review & Editing, S.P.H. and K.B.; Funding Acquisition, S.P.H. and K.B.; Supervision, S.P.H. and K.B.

DECLARATION OF INTERESTS

The authors declare no competing interests.

Received: October 17, 2018
Revised: April 1, 2019
Accepted: May 15, 2019
Published: June 11, 2019

REFERENCES

Arima, S., Nishiyama, K., Ko, T., Arima, Y., Hakozaki, Y., Sugihara, K., Koseki, H., Uchijima, Y., Kurihara, Y., and Kurihara, H. (2011). Angiogenic morphogenesis driven by dynamic and heterogeneous collective endothelial cell movement. Development *138*, 4763–4776.

Benedito, R., Rocha, S.F., Woeste, M., Zamykal, M., Radtke, F., Casanovas, O., Duarte, A., Pytowski, B., and Adams, R.H. (2012). Notch-dependent VEGFR3 upregulation allows angiogenesis without VEGF-VEGFR2 signalling. Nature *484*, 110–114.

Bentley, K., and Chakravartula, S. (2017). The temporal basis of angiogenesis. Philos. Trans. R. Soc. Lond. B Biol. Sci. *372*, 20150522.

Bentley, K., Gerhardt, H., and Bates, P.A. (2008). Agent-based simulation of notch-mediated tip cell selection in angiogenic sprout initialisation. J. Theor. Biol. *250*, 25–36.

Bentley, K., Mariggi, G., Gerhardt, H., and Bates, P.A. (2009). Tipping the balance: robustness of tip cell selection, migration and fusion in angiogenesis. PLoS Comput. Biol. *5*, e1000549.

Bentley, K., Franco, C.A., Philippides, A., Blanco, R., Dierkes, M., Gebala, V., Stanchi, F., Jones, M., Aspalter, I.M., Cagna, G., et al. (2014a). The role of differential VE-cadherin dynamics in cell rearrangement during angiogenesis. Nat. Cell Biol. *16*, 309–321.

Bentley, K., Harrington, K., and Regan, E. (2014b). Can active perception generate bistability? Heterogeneous collective dynamics and vascular patterning. Artificial Life Conference Proceedings *26*, 328–335.

Blum, Y., Belting, H.-G., Ellertsdottir, E., Herwig, L., Lüders, F., and Affolter, M. (2008). Complex cell rearrangements during intersegmental vessel sprouting and vessel fusion in the zebrafish embryo. Dev. Biol. *316*, 312–322.

Brandman, O., and Meyer, T. (2008). Feedback loops shape cellular signals in space and time. Science *322*, 390–395.

Carmeliet, P., and Jain, R.K. (2011). Molecular mechanisms and clinical applications of angiogenesis. Nature *473*, 298–307.

Cermak, T., Doyle, E.L., Christian, M., Wang, L., Zhang, Y., Schmidt, C., Baller, J.A., Somia, N.V., Bogdanove, A.J., and Voytas, D.F. (2011). Efficient design and assembly of custom TALEN and other TAL effector-based constructs for DNA targeting. Nucleic Acids Res. *39*, e82.

Costa, G., Harrington, K.I., Lovegrove, H.E., Page, D.J., Chakravartula, S., Bentley, K., and Herbert, S.P. (2016). Asymmetric division coordinates collective cell migration in angiogenesis. Nat. Cell Biol. *18*, 1292–1301.

Covassin, L.D., Villefranc, J.A., Kacergis, M.C., Weinstein, B.M., and Lawson, N.D. (2006). Distinct genetic interactions between multiple Vegf receptors are required for development of different blood vessel types in zebrafish. Proc. Natl. Acad. Sci. USA *103*, 6554–6559.

Fish, J.E., Cantu Gutierrez, M., Dang, L.T., Khyzha, N., Chen, Z., Veitch, S., Cheng, H.S., Khor, M., Antounians, L., Njock, M.-S., et al. (2017). Dynamic regulation of VEGF-inducible genes by an ERK-ERG-p300 transcriptional network. Development *144*, 2428–2444.

Freeman, M. (2000). Feedback control of intercellular signalling in development. Nature *408*, 313–319.

Gao, H., Chakraborty, G., Zhang, Z., Akalay, I., Gadiya, M., Gao, Y., Sinha, S., Hu, J., Jiang, C., Akram, M., et al. (2016). Multi-organ site metastatic reactivation mediated by non-canonical discoidin domain receptor 1 signaling. Cell *166*, 47–62.

Gerhardt, H., Golding, M., Fruttiger, M., Ruhrberg, C., Lundkvist, A., Abramsson, A., Jeltsch, M., Mitchell, C., Alitalo, K., Shima, D., and Betsholtz, C. (2003). VEGF guides angiogenic sprouting utilizing endothelial tip cell filopodia. J. Cell Biol. *161*, 1163–1177.

Hellström, M., Phng, L.-K., Hofmann, J.J., Wallgard, E., Coultas, L., Lindblom, P., Alva, J., Nilsson, A.-K., Karlsson, L., Gaiano, N., et al. (2007). Dll4 signalling through Notch1 regulates formation of tip cells during angiogenesis. Nature *445*, 776–780.

Herbert, S.P., and Stainier, D.Y.R. (2011). Molecular control of endothelial cell behaviour during blood vessel morphogenesis. Nat. Rev. Mol. Cell Biol. *12*, 551–564.

Herbert, S.P., Cheung, J.Y.M., and Stainier, D.Y.R. (2012). Determination of endothelial stalk versus tip cell potential during angiogenesis by H2.0-like homeobox-1. Curr. Biol. *22*, 1789–1794.

Hogan, B.M., Herpers, R., Witte, M., Heloterä, H., Alitalo, K., Duckers, H.J., and Schulte-Merker, S. (2009). Vegfc/Flt4 signalling is suppressed by Dll4 in developing zebrafish intersegmental arteries. Development *136*, 4001–4009.

Jakobsson, L., Franco, C.A., Bentley, K., Collins, R.T., Ponsioen, B., Aspalter, I.M., Rosewell, I., Busse, M., Thurston, G., Medvinsky, A., et al. (2010). Endothelial cells dynamically compete for the tip cell position during angiogenic sprouting. Nat. Cell Biol. *12*, 943–953.

Jao, L.-E., Wente, S.R., and Chen, W. (2013). Efficient multiplex biallelic zebrafish genome editing using a CRISPR nuclease system. Proc. Natl. Acad. Sci. USA *110*, 13904–13909.

Jin, S.W., Beis, D., Mitchell, T., Chen, J.N., and Stainier, D.Y. (2005). Cellular and molecular analyses of vascular tube and lumen formation in zebrafish. Development *132*, 5199–5209.

Jung, O., Choi, Y.-J., Kwak, T.K., Kang, M., Lee, M.-S., Ryu, J., Kim, H.-J., and Lee, J.W. (2013). The COOH-terminus of TM4SF5 in hepatoma cell lines regulates c-Src to form invasive protrusions via EGFR Tyr845 phosphorylation. Biochim. Biophys. Acta *1833*, 629–642.

Krueger, J., Liu, D., Scholz, K., Zimmer, A., Shi, Y., Klein, C., Siekmann, A., Schulte-Merker, S., Cudmore, M., Ahmed, A., and le Noble, F. (2011). Flt1 acts as a negative regulator of tip cell formation and branching morphogenesis in the zebrafish embryo. Development *138*, 2111–2120.

Kur, E., Kim, J., Tata, A., Comin, C.H., Harrington, K.I., Costa, Lda.F., Bentley, K., and Gu, C. (2016). Temporal modulation of collective cell behavior controls vascular network topology. eLife *5*, a001875.

Larkin, M.A., Blackshields, G., Brown, N.P., Chenna, R., McGettigan, P.A., McWilliam, H., Valentin, F., Wallace, I.M., Wilm, A., Lopez, R., et al. (2007). Clustal W and Clustal X version 2.0. Bioinformatics *23*, 2947–2948.

Leslie, J.D., Ariza-McNaughton, L., Bermange, A.L., McAdow, R., Johnson, S.L., and Lewis, J. (2007). Endothelial signalling by the Notch ligand Delta-like 4 restricts angiogenesis. Development *134*, 839–844.

Lobov, I.B., Renard, R.A., Papadopoulos, N., Gale, N.W., Thurston, G., Yancopoulos, G.D., and Wiegand, S.J. (2007). Delta-like ligand 4 (Dll4) is induced by VEGF as a negative regulator of angiogenic sprouting. Proc. Natl. Acad. Sci. USA *104*, 3219–3224.

Matsuda, M., Koga, M., Woltjen, K., Nishida, E., and Ebisuya, M. (2015). Synthetic lateral inhibition governs cell-type bifurcation with robust ratios. Nat. Commun. *6*, 6195.

Nagasawa-Masuda, A., and Terai, K. (2016). ERK activation in endothelial cells is a novel marker during neovasculogenesis. Genes Cells *21*, 1164–1175.

Page, R.D. (1996). TreeView: an application to display phylogenetic trees on personal computers. Comput. Appl. Biosci. *12*, 357–358.

Potente, M., Gerhardt, H., and Carmeliet, P. (2011). Basic and therapeutic aspects of angiogenesis. Cell *146*, 873–887.

Ran, F.A., Hsu, P.D., Wright, J., Agarwala, V., Scott, D.A., and Zhang, F. (2013). Genome engineering using the CRISPR-Cas9 system. Nat. Protoc. *8*, 2281–2308.

Reischauer, S., Stone, O.A., Villasenor, A., Chi, N., Jin, S.-W., Martin, M., Lee, M.T., Fukuda, N., Marass, M., Witty, A., et al. (2016). Cloche is a bHLH-PAS transcription factor that drives haemato-vascular specification. Nature *535*, 294–298.

Rocha, S.F., Schiller, M., Jing, D., Li, H., Butz, S., Vestweber, D., Biljes, D., Drexler, H.C.A., Nieminen-Kelhä, M., Vajkoczy, P., et al. (2014). Esm1 modulates endothelial tip cell behavior and vascular permeability by enhancing VEGF bioavailability. Circ. Res. *115*, 581–590.

Roukens, M.G., Alloul-Ramdhani, M., Baan, B., Kobayashi, K., Peterson-Maduro, J., van Dam, H., Schulte-Merker, S., and Baker, D.A. (2010). Control of endothelial sprouting by a Tel-CtBP complex. Nat. Cell Biol. *12*, 933–942.

Ruhrberg, C., Gerhardt, H., Golding, M., Watson, R., Ioannidou, S., Fujisawa, H., Betsholtz, C., and Shima, D.T. (2002). Spatially restricted patterning cues provided by heparin-binding VEGF-A control blood vessel branching morphogenesis. Genes Dev. *16*, 2684–2698.

Sacilotto, N., Chouliaras, K.M., Nikitenko, L.L., Lu, Y.W., Fritzsche, M., Wallace, M.D., Nornes, S., García-Moreno, F., Payne, S., Bridges, E., et al. (2016). MEF2 transcription factors are key regulators of sprouting angiogenesis. Genes Dev. *30*, 2297–2309.

Shih, S.C., Zukauskas, A., Li, D., Liu, G., Ang, L.H., Nagy, J.A., Brown, L.F., and Dvorak, H.F. (2009). The L6 protein TM4SF1 is critical for endothelial cell function and tumor angiogenesis. Cancer Res. *69*, 3272–3277.

Shin, M., Beane, T., Quillien, A., Male, I., Zhu, L.J., and Lawson, N.D. (2016). Vegfa signals through ERK to promote angiogenesis, but not artery differentiation. Development *143*, 3796–3805.

Siekmann, A.F., and Lawson, N.D. (2007). Notch signalling limits angiogenic cell behaviour in developing zebrafish arteries. Nature *445*, 781–784.

Sprinzak, D., Lakhanpal, A., Lebon, L., Santat, L.A., Fontes, M.E., Anderson, G.A., Garcia-Ojalvo, J., and Elowitz, M.B. (2010). Cis interactions between Notch and Delta generate mutually exclusive signalling states. Nature *465*, 86–90.

Stamatakis, A. (2014). RAxML version 8: a tool for phylogenetic analysis and post-analysis of large phylogenies. Bioinformatics *30*, 1312–1313.

Suchting, S., Freitas, C., le Noble, F., Benedito, R., Bréant, C., Duarte, A., and Eichmann, A. (2007). The Notch ligand Delta-like 4 negatively regulates endothelial tip cell formation and vessel branching. Proc. Natl. Acad. Sci. USA *104*, 3225–3230.

Tang, R., Dodd, A., Lai, D., McNabb, W.C., and Love, D.R. (2007). Validation of zebrafish (Danio rerio) reference genes for quantitative real-time RT-PCR normalization. Acta Biochim. Biophys. Sin. (Shanghai) *39*, 384–390.

Thisse, C., and Thisse, B. (2008). High-resolution in situ hybridization to whole-mount zebrafish embryos. Nat. Protoc. *3*, 59–69.

Ubezio, B., Blanco, R.A., Geudens, I., Stanchi, F., Mathivet, T., Jones, M.L., Ragab, A., Bentley, K., and Gerhardt, H. (2016). Synchronization of endothelial Dll4-Notch dynamics switch blood vessels from branching to expansion. eLife *5*, 186.

Venkatraman, L., Regan, E.R., and Bentley, K. (2016). Time to decide? Dynamical analysis predicts partial tip/stalk patterning states arise during angiogenesis. PLoS ONE *11*, e0166489.

Villefranc, J.A., Nicoli, S., Bentley, K., Jeltsch, M., Zarkada, G., Moore, J.C., Gerhardt, H., Alitalo, K., and Lawson, N.D. (2013). A truncation allele in *vascular endothelial growth factor c* reveals distinct modes of signaling during lymphatic and vascular development. Development *140*, 1497–1506.

Zarkada, G., Heinolainen, K., Makinen, T., Kubota, Y., and Alitalo, K. (2015). VEGFR3 does not sustain retinal angiogenesis without VEGFR2. Proc. Natl. Acad. Sci. USA *112*, 761–766.

Zukauskas, A., Merley, A., Li, D., Ang, L.-H., Sciuto, T.E., Salman, S., Dvorak, A.M., Dvorak, H.F., and Jaminet, S.-C.S. (2011). TM4SF1: a tetraspanin-like protein necessary for nanopodia formation and endothelial cell migration. Angiogenesis *14*, 345–354.

STAR★METHODS

KEY RESOURCES TABLE

REAGENT or RESOURCE	SOURCE	IDENTIFIER
Antibodies		
rabbit anti-ERK1/2	Cell Signaling	Cat# 4695; RRID: AB_390779
rabbit anti-pERK1/2	Cell Signaling	Cat# 4377; RRID: AB_331775
rabbit anti-pERK1/2	Cell Signaling	Cat# 4370; RRID: AB_2315112
goat anti-rabbit IgG-HRP	Cell Signaling	Cat# 7074; RRID: AB_2099233
Chemicals, Peptides, and Recombinant Proteins		
SU5416	Sigma Aldrich	Cat# S8442
ZM323881	R&D systems	Cat# 2475
5-hydroxyurea	Sigma Aldrich	Cat# H8627
aphidicolin	Sigma Aldrich	Cat# 89458
Ki8751	TOCRIS	Cat# 2542
DAPT	Sigma Aldrich	Cat# D5942
DBZ	TOCRIS	Cat# 4489
Blocking reagent	Sigma-aldrich	Cat# 11096176001
TrypLE	Invitrogen	Cat# 12604013
Endothelial Cell Growth Medium 2 KIT	Promocell	Cat# C-22111
Critical Commercial Assays		
TSA Plus Cy3 Fluorescence System	Perkin Elmer	Cat# NEL 74400KT
Meltdoctor HRM Mastermix	ThermoFisher	Cat# 4415440
SYBR Green Mastermix	ThermoFisher	Cat# 4309155
RNAqueous-Micro kit	ThermoFisher	Cat# AM1931
GeneFECTOR reagent	Venn-Nova	Cat# R-0001-01
Deposited Data		
Raw and analyzed microarray datasets	(Herbert et. al. 2012) and this paper	GEO: GSE130889
Experimental Models: Cell Lines		
Human: pooled primary HUVEC	Promocell	Cat# C-12203
Experimental Models: Organisms/Strains		
Zebrafish: *npas4l*^{s5} mutant	Reischauer et al., 2016	ZFIN ID: ZDB-ALT-010426-6
Zebrafish: *Tg(kdrl:GFP)*^{s843}	Jin et al., 2005	ZFIN ID: ZDB-ALT-050916-14
Zebrafish: *Tg(kdrl:nlsEGFP)*^{zf109}	Blum et. al., 2008	ZFIN: ZDB-ALT-081105-1
Zebrafish: *tm4sf18* exon1 mutant	This paper	N/A
Zebrafish: *tm4sf18* exon2 mutant	This paper	N/A
Oligonucleotides		
Control morpholino oligonucleotide: 5′- CCTCTTACCTCA GTTACAATTTATA –3′	Gene Tools	Cat# 2934999000
flt1 morpholino oligonucleotide: 5′- ATATCGAACATTCTC TTGGTCTTGC –3′	Gene Tools	ZFIN ID: ZDB-MRPHLNO-110531-3
dll4 morpholino oligonucleotide: 5′- GTTCGAGCTTACCG GCCACCCAAAG –3′	Gene Tools	ZFIN ID: ZDB-MRPHLNO-070509-1
Primers for qPCR, see Table S1	This paper	N/A
TM4SF1 siGENOME SMARTpool siRNA	Dharmacon	M-010610-01
Control siGENOME non target pool siRNA	Dharmacon	D-001206-13
Primers for genotyping, see Table S1	This paper	N/A

(*Continued on next page*)

Continued

REAGENT or RESOURCE	SOURCE	IDENTIFIER
CRISPR/Cas9 targeting site sequence: *tm4sf18*: CCTGTG TGTTCCTGGGAATG	Eurogentec	N/A
TALEN targeting site sequences: *tm4sf18*: TGTGCTCTAC AGGATTTGCC	Eurogentec	N/A
GCCCTGGTCCCTCTCGCCA		
Recombinant DNA		
pCR-Blunt II-TOPO	Invitrogen	Cat# K280002
pCR-Blunt II-TOPO-tm4sf18	This paper	N/A
Software and Algorithms		
memAgent-spring model of lateral inhibition	This paper	N/A
ODE model of lateral inhibition	This paper	N/A

CONTACT FOR REAGENT AND RESOURCE SHARING

Further information and requests for resources and reagents should be directed to and will be fulfilled by the Lead Contact, Shane Herbert (shane.herbert@manchester.ac.uk).

EXPERIMENTAL MODEL AND SUBJECT DETAILS

Animals

Zebrafish embryos, larvae, and adults were grown and maintained according to UK Home Office regulation guidelines and all studies were approved by the University of Manchester Ethical Review Board. Zebrafish strains were maintained at pH 7.4, a temperature of 28°C and exposed to 14 h light and 10 h dark cycles. Zebrafish from 6 to 12 months of age were used for breeding. Following breeding, embryos were transferred to Petri dishes containing E3 media and incubated at 28°C until required for experiments. Embryos used for experiments were less than 4 days post fertilization, a stage at which sex cannot be readily determined and is unlikely to influence the biological processes under study. Previously described zebrafish lines used in this study were the *npas4l*^{s5} mutant, *Tg(kdrl:GFP)*^{s843} strain and *Tg(kdrl:nlsEGFP)*^{zf109} strain (Blum et al., 2008; Jin, et al., 2005; Reischauer et al., 2016).

Primary cell culture

Pooled primary Human umbilical vein endothelial cells (HUVECs) were purchased from Promocell (C-12203). Cells were cultured in endothelial cell basal medium supplemented with endothelial cell growth medium 2 kit (Promocell; C-22111) at 5% CO2 and a temperature of 37°C. All cells were grown on 0.1% (w/v) gelatin-coated cultureware and were not used in excess of four passages.

METHOD DETAILS

Time-lapse imaging

Confocal microscopy of live *Tg(kdrl:nlsEGFP)*^{zf109} embryos was performed as previously described (Costa et al., 2016; Herbert et al., 2012). Briefly, embryos were mounted in 1% low-melt agarose in glass bottom dishes, which were subsequently filled with media supplemented with 0.0045% 1-Phenyl-2-thiourea and 0.1% tricaine. Embryos were imaged using a 20x dipping objectives on a Zeiss LSM 700 confocal microscope. Embryos were maintained at 28°C and stacks were recorded at every 0.3 h. Tracking of cell motility was performed in ImageJ using the manual tracking plugin. All cell tracking recordings were normalized at each time point relative to the position of the dorsal aorta to account for any dorsal or ventral drift of embryos during imaging.

Morpholino oligonucleotide (MO) injections

To knock down gene expression, embryos were injected at the one-cell stage with 5 ng control MO, 5 ng *dll4* MO or 1-2 ng of *flt1* MO. The control MO targets the human beta-globin intron mutation underpinning beta-thalassemia. As such, this MO has no phenotypic effect in zebrafish and other model systems, except human beta-thalassemic hematopoietic cells. MO sequences were:

5′- CCTCTTACCTCAGTTACAATTTATA −3′ (control)
5′- ATATCGAACATTCTCTTGGTCTTGC – 3′ (*flt1*) (Krueger et al., 2011)
5′- GTTCGAGCTTACCGGCCACCCAAAG −3′ (*dll4*) (Siekmann and Lawson, 2007).

All MOs were purchased from Gene Tools.

Pharmacological treatments

Embryos were manually dechorionated and incubated with compounds from 22 hpf (unless otherwise stated). The following compounds were used in this study: SU5416 (0.3 μM, 2.5 μM), ZM323881 (40 nM, 80 nM and 160 nM), 5-hydroxyurea (150 μM), aphidicolin (20 mM), Ki8751 (0.5 μM), DAPT (100 μM) and DBZ (2 μM).

Simulations with the memAgent-spring model (MSM)

The MSM model has been well validated against *in-vivo* mouse and zebrafish ISV data in previous studies of collective cell dynamics during of Vegf-Notch-mediated tip cell selection, so it made a good choice for simulating the dynamics within the time window observed *in-vivo*. In this model, the endothelial cell outer membrane is represented at a subcellular level by a collection of individual computational agents ('memAgents') connected by springs following Hooke's law, which represents the actin cortex beneath. The MSM allows subcellular level rules to generate localized responses of individual memAgents on the cell surface and complex cell shape changes during cell migration.

Model initialization and parameterization

The model was initialized with 8 cells in a row, one per vessel cross section (See Figure S1d), representing a collection of endothelial cells in the DA competing to sprout into the ISV space above (represented very simply here as just a fixed vegf gradient extending into the y axis above the horizontal row of cells). All parameters were kept the same as previously published (Bentley et al., 2008, 2009; Kur et al., 2016) except those being varied to match the experimental conditions here, described below.

The model was run 100 times for a maximum of 200 timesteps under a range of *vegf* and *flt1* inhibition conditions to see if a single early time window during selection might also generate fewer or more cells being selected by those times as seen *in-vivo* for some values of the respective vegf perturbation conditions. VEGF – vegfr activation $(V_m{}')$ of the vegfr (*V*) in a given memAgent *m* in the model is encapsulated by Equation 1 (fully described in (Bentley et al., 2008)) below:

$$V_m^{'} = V_{sink} V_m M_{tot} \Big/ V_{max} \sum_{n=1}^{26} E_n .\ VEGF \tag{1}$$

Where, *Vsink* (normally set to 9*)* is a fixed value which acts as a sink (mimicking *flt1*) reducing the amount of available *VEGF* in the 26 neighboring environmental grid sites ($E_n.VEGF$) surrounding that memAgent *m* (as the model runs on the 3D gridded lattice) for binding to its main vegf receptors V_m (only the main receptor V is able to trigger cell migration and Dll4 upregulation in the cells). M_{tot} is the total number of memAgents currently comprising the cell, and V_{max} is the maximum number of receptors the cell can have. This is the only equation that was varied here, by simply reducing the levels of *VEGF* (to model vegf inhibition) and *Vsink* (to model *flt1* loss) respectively.

ODE model construction & simulation

Interaction between two ECs have been captured using coupled ordinary differential equations and the two-cell model previously described (Venkatraman et al., 2016). Reactions for the ordinary differential equations of the two-cell model were written following mass-action kinetics. Details of model construction, list of ODEs, reaction equations and parameters can be found in (Venkatraman et al., 2016). Positive-feedback between VEGF and a VEGF-induced/activated factor (*P*) is captured using Equation 2 below;

$$V = V * (1 + k_6 * P^n) \tag{2}$$

Where, k_6 is the positive-feedback rate of *P* production and *n* captures the non-linearity of signaling between VEGF (*V*) sensing and *P*. In this model *n* is set at 2 to reflect presumed cooperativity between at least two pathways that lead to positive-feedback. This is consistent with observations that poor decision making and selection of ECs is observed when feedback levels are set to 0, whereas a less severe phenotype is observed upon *tm4sf18* mutation in zebrafish. Hence, these data hint that at least two positive-feedback pathways cooperatively operate *in-vivo*. Model simulations were performed using ODE15s solver in MATLAB2013b (https://www.mathworks.com). All steady state analysis of the ODE model was carried out using the AUTO bifurcation toolbox in XPPAUT (http://www.math.pitt.edu/∼bard/xpp/xpp.html).

Isolation of zebrafish ECs and transcriptome analyses

Previously published microarray datasets of isolated zebrafish ECs (Herbert et al., 2012) were re-analyzed in this study and are deposited online (GEO: GSE130889). Briefly, for flow cytometry-mediated isolation of zebrafish ECs, *Tg(kdrl:GFP)*^{s843} embryos were dissected and trunks collected in ice cold Ca^{2+}/Mg^{2+}-free Hank's buffered salt solution (HBSS), washed four times in 1 mL ice cold Ca^{2+}/Mg^{2+}-free HBSS and dissociated in 2 mL TrypLE (Invitrogen) at 27.5°C for 30 min with regular agitation. Dissociation was inactivated upon addition of 100 μl fetal bovine serum (FBS). Dissociated cells were subsequently isolated by centrifugation, re-suspended in 5 mL Ca^{2+}/Mg^{2+}-containing HBSS (with 5% FBS) and passed through 40 μm filters. ECs were collected upon re-centrifugation of dissociated cells, re-suspension in 0.5 mL Ca^{2+}/Mg^{2+}-containing HBSS (with 5% FBS) and FACS isolation of the *kdrl*:GFP-positive cell population directly into lysis buffer. Total RNA was isolated using the RNAqueous-Micro kit (ThermoFisher). Complementary DNAs were amplified, labeled with Cy3 (from DMSO-treated embryos) or Cy5 (chemical-treated embryos) and hybridized to the Agilent Zebrafish Gene Expression Microarray (V2) by Mogene Lc. The extracted data were normalized and quality

controlled using GeneSpring GX software (Agilent). All probes with a green processed signal below 100 were considered as background. Cut-offs used to identify Vegfr-regulated transcripts were: ratio SU5416 versus DMSO = < 0.4; ratio DAPT versus DMSO = > 1.5; ratio SU5416 + DAPT versus DMSO = < 1. Of the 10 hit transcripts identified, we prioritized *tm4sf18* for genetic functional studies as *tm4sf18* exists as a single gene in zebrafish and does not exist as multiple gene paralogs that potentially exhibit functional redundancy. As such, genetic functional studies of *tm4sf18* were considered highly tractable versus studies of other identified transcripts.

Quantitative real-time PCR (qPCR)

Zebrafish embryo or HUVEC cDNA samples were diluted to a concentration of 50 ng/μl with dH_20. Each qPCR reaction was prepared in triplicate in a 48 or 96-well plate with each well consisting of 0.2 μM each forward and reverse primer, 50ng cDNA and SYBR Green Mastermix (ThermoFisher). Reactions were run on an Eco Real-Time PCR System (Illumina) or Step One Plus Real-time PCR System (Applied Biosystems) alongside negative controls. qPCR data was analyzed by the $\Delta\Delta C_T$ method and expression normalized to *β-actin* and *ef1α* (zebrafish) or *GAPDH* (human). A relative quantification of gene expression was then determined using the formula $2^{-\Delta\Delta CT}$. Primers used for qPCR amplification were:

zebrafish *β actin* forward: 5′-CGAGCTGTCTTCCCATCCA-3′
zebrafish *β actin* reverse: 5′-TCACCAACGTAGCTGTCTTTCTG-3′ (Tang et al., 2007)
zebrafish *dll4* forward: 5′-TGGCCAGTTATCCTGTCTCC-3′
zebrafish *dll4* reverse: 5′-CTCACTGCATCCCTCCAGAC-3′ (Roukens et al., 2010)
zebrafish *ef1α* forward: 5′-CTGGAGGCCAGCTCAAACAT-3′
zebrafish *ef1α* reverse: 5′-ATCAAGAAGAGTAGTACCGCTAGCATTAC-3′ (Tang et al., 2007)
zebrafish *flt4* forward: 5′-CTGTCGGATTTGGATTGGGA-3′
zebrafish *flt4* reverse: 5′-GGTGGACTCATAGAAAACCCATTC-3′ (Covassin et al., 2006)
zebrafish *kdrl* forward: 5′-ACTTTGAGTGGGAGTTTCATAAGGA-3′
zebrafish *kdrl* reverse: 5′-TTGGACCGGTGTGGTGCTA-3′ (Covassin et al., 2006)
zebrafish *tm4sf18* forward: 5′-CTGGATACTGCTTCCTGATCTC-3′
zebrafish *tm4sf18* reverse: 5′-CAAACAGATACCGTCCCTCAT-3′
human *GAPDH* forward: 5′-TGCACCACCAACTGCTTAGC-3′
human *GAPDH* reverse: 5′-GGCATGGACTGTGGTCATGAG-3′
human *TM4SF1* forward: 5′-CTTCGTGTGGTTCTTTTCTG-3′
human *TM4SF1* reverse: 5′-ATCGTTTGCCACAGTTTTC-3′

Cloning of tm4sf18 and whole-mount *in situ* hybridization

The zebrafish tm4sf18 *in situ* hybridization construct was generated by PCR amplification of the tm4sf18 ORF from cDNA and cloning of this fragment into pCR-Blunt II-TOPO (Invitrogen). For probe generation, pCR-Blunt II-TOPO tm4sf18 was linearized with EcoRV at 37°C for 3 h and T7 was used for transcription. For whole-mount *in situ* hybridization, embryos were fixed in 4% paraformaldehyde (PFA) overnight at 4°C and processed as described previously (Thisse and Thisse, 2008).

siRNA-mediated gene knockdown

For gene knockdown, HUVECs were seeded at 0.2 × 106 cells/well in 6-well plates and transfected with TM4SF1 siGENOME SMARTpool siRNA or Control siRNA (Thermo Scientific) using the GeneFECTOR reagent, (Venn-Nova), as per manufacturer's instructions. Cells were processed for RNA extraction 48 h after transfection.

Immunoblotting

HUVECs were lysed in RIPA lysis buffer and prepared for immunoblotting in laemmli buffer. Western blotting was performed using Biorad mini-protean gels and transfer kits according to manufacturers' instructions. Nitrocellulose membranes were incubated with antibodies in the respective blocking buffers according the manufacturers' recommendations of each antibody. The antibodies used for immunoblotting in this study were; rabbit anti-ERK1/2 (Cell Signaling; #4695) and rabbit anti-pERK1/2 (Cell Signaling; #4377). Membrane bound antibodies were detected by ECL (ThermoScientific).

Phylogenetic analysis

For phylogenetic analysis of the TM4SF1/4/18 protein family, the NCBI Reference Sequences of TM4SF1, TM4SF4, TM4SF18 proteins of each species were used respectively: human (*Homo sapiens*; NP_055035.1, NP_004608.1, NP_620141.1), mouse (*Mus musculus*; NP_032562.1, NP_663514.2), chicken (*Gallus gallus*; NP_001264407.1, XP_001234023.1, XP_001234069.1), turtle (*Pelodiscus sinensis*; XP_006115992.1, XP_006115993.1, XP_006115991.1), frog (*Xenopus tropicalis*; XP_002937372.1, NP_988958.1, XP_002937374.1), medaka (*Oryzias latipes*; XP_004068091.1, XP_004068090.1), zebrafish (*Danio rerio*; NP_001003489.1, NP_001038487.2), fugu (*Takifugu rubripes*; XP_003974429.1, UniProt H2VCB8), tetraodon (*Tetraodon nigroviridis*; CAF90631.1, CAF90632.1) and elephant shark (*Callorhinchus milii*; XP_007900629.1). Human TM4SF5 protein (NP_003954.2) was used as an out-group for our phylogenetic analysis.

The TM4SF1/4/18 amino acid sequences from all 10 species were aligned using Clustal X2 (Larkin et al., 2007). The sequences were then manually trimmed of all sites that were not unambiguously aligned. Phylogenetic analysis of amino acid sequences was first performed using NL method implemented in ClustalX2, with outputs displayed using TreeView (Page, 1996). Confidence in the phylogeny was assessed by bootstrap re-sampling of the data. For ML tree, the JTT model of protein evolution was used in RAxML (Stamatakis, 2014) with the proportion of invariable sites and gamma parameter estimated from the data, four categories of between-site rate variation; 100 bootstraps were used in the primary ML tree (final ML optimization likelihood: −4945.841564).

Gene editing

TALENs were designed and constructed to target exon-1 of zebrafish *tm4sf18* using online tools (https://tale-nt.cac.cornell.edu/node/add/talen) and as previously described using the Golden Gate method (Cermak et al., 2011). The target sequences chosen for the forward and reverse TALENs were 5′–TGTGCTCTACAGGATTTGCC-3′ and 5′-GCCCTGGTCCCTCTCGCCA-3′, respectively. Repeat Variable Diresidue (RVD) sequence for the forward TALEN was: NH NG NH HD NG HD NG NI HD NI NH NH NI NG NG NG NH HD HD. RVD sequence for the reverse TALEN was: NH NH HD NH NI NH NI NH NH NH NI HD HD NI NH NH NH HD. Length of the spacer DNA between TALEN-binding sequences was 15 base pairs. 100 pg of both forward and reverse TALEN mRNA was co-injected into the single cell of zebrafish embryos. At around 24-72 hpf, genomic DNA was extracted from individual embryos and somatic lesions confirmed by high resolution melt (HRM) using Meltdoctor HRM Mastermix (ThermoFisher) and the following primers:

tm4sf18 TALEN forward 5′-CTGTTTTCTCCCCCACACAC-3′
tm4sf18 TALEN reverse 5′-TACTCACAGCCAGACCACCA-3′

The CRISPR target site within exon-2 of *tm4sf18* (5′-CCTGTGTGTTCCTGGGAATG-3′) was identified as previously (Ran et al., 2013). gRNA and *nls-zCas9-nls* RNA were generated as previously (Jao et al., 2013). 30-100 ng of gRNA and 100-150 ng of *nls-zCas9-nls* RNA were co-injected into single cell stage embryos mixed with a phenol red tracer. At around 24-72 hpf, genomic DNA was extracted from individual embryos and somatic lesions confirmed by HRM (as above) using the following primers:

tm4sf18 CRISPR forward 5′- CATCAGTCTTTGCAGCGAGA −3′
tm4sf18 CRISPR reverse 5′- TGTAGCATATCCCAACACTCAC −3′

pErk immunostaining

Whole-mount immunostaining for pErk was performed as previously described (Costa et al., 2016). Briefly, *Tg(kdrl:nlsEGFP)*^{zf109} embryos were fixed in PFA overnight prior to washing in 100% MeOH, incubation with 3% $H_2 0_2$ in MeOH on ice for 60 min and further 100% MeOH washes. Embryos were then stored at −20°C for 2 days in MeOH before equilibration with PBT (PBS, 0.1% Tween-20) washes and cryoprotected in 30% sucrose in PBT overnight at 4°C. The next day embryos were equilibrated in PBT, incubated with 150 mM Tris-HCl (pH 9.0) for 5 min and then heated to 70°C for 15 min. Embryos were then washed with PBT and then twice with dH_2O for 5 min. Water was then removed prior to addition of ice-cold acetone for 20 min at −20°C. Acetone was removed prior to PBT washes, one TBST (TBS, 0.1% Tween-20, 0.1% Triton X-100) wash and incubation overnight at 4°C with block solution (TBST, 1% BSA, 10% goat serum). The next day embryos were then incubated with anti-phospho-ERK1/2 antibody (1:250, Cell Signaling; #4370) in blocking buffer overnight at 4°C. Washes in TBST at room temperature were followed by a wash in Maleic buffer (150 mM Maleic acid, 100 mM NaCl, 0.001% Tween-20, pH 7.4) for 30 min. Embryos were then blocked in 2% blocking reagent (Sigma-Aldrich) in Maleic buffer for 3 h at room temperature prior to incubation with goat anti-rabbit IgG-HRP (1:1000) in 2% blocking reagent in Maleic buffer overnight at 4°C. Embryos were then washed in Maleic buffer and then PBS at room temperature prior to incubation with 50 μl amplification diluent with 1 μl Tyramide-Cy3 (Perkin Elmer) for 3 h at room temperature in the dark. Embryos were finally washed over several days in TBST at room temperature. Levels of pErk were quantified as the mean nuclear Cy3 fluorescence intensity using ImageJ.

QUANTIFICATION AND STATISTICAL ANALYSIS

All statistical analyses were performed using GraphPad Prism 6.0 or Microsoft Excel software. Statistical significance was assessed using either unpaired two-tailed Student’s t tests or two-way ANOVA tests, as reported in the figure legends. Results are presented as mean ± SEM. For all analyses, $p < 0.05$ was considered statistically significant. No data points or subjects were excluded from analyses. No statistical method was used to estimate sample size, but sample sizes used were consistent with those employed in the field.

DATA AND SOFTWARE AVAILABLITY

The MSM and ODE models have been previously published, but all new code generated to facilitate studies of positive-feedback are available upon request. Microarray datasets analyzed in this study are deposited online (GEO: GSE130889).

Cell Reports, Volume *27*

Supplemental Information

Positive Feedback Defines the Timing, Magnitude, and Robustness of Angiogenesis

Donna J. Page, Raphael Thuret, Lakshmi Venkatraman, Tokiharu Takahashi, Katie Bentley, and Shane P. Herbert

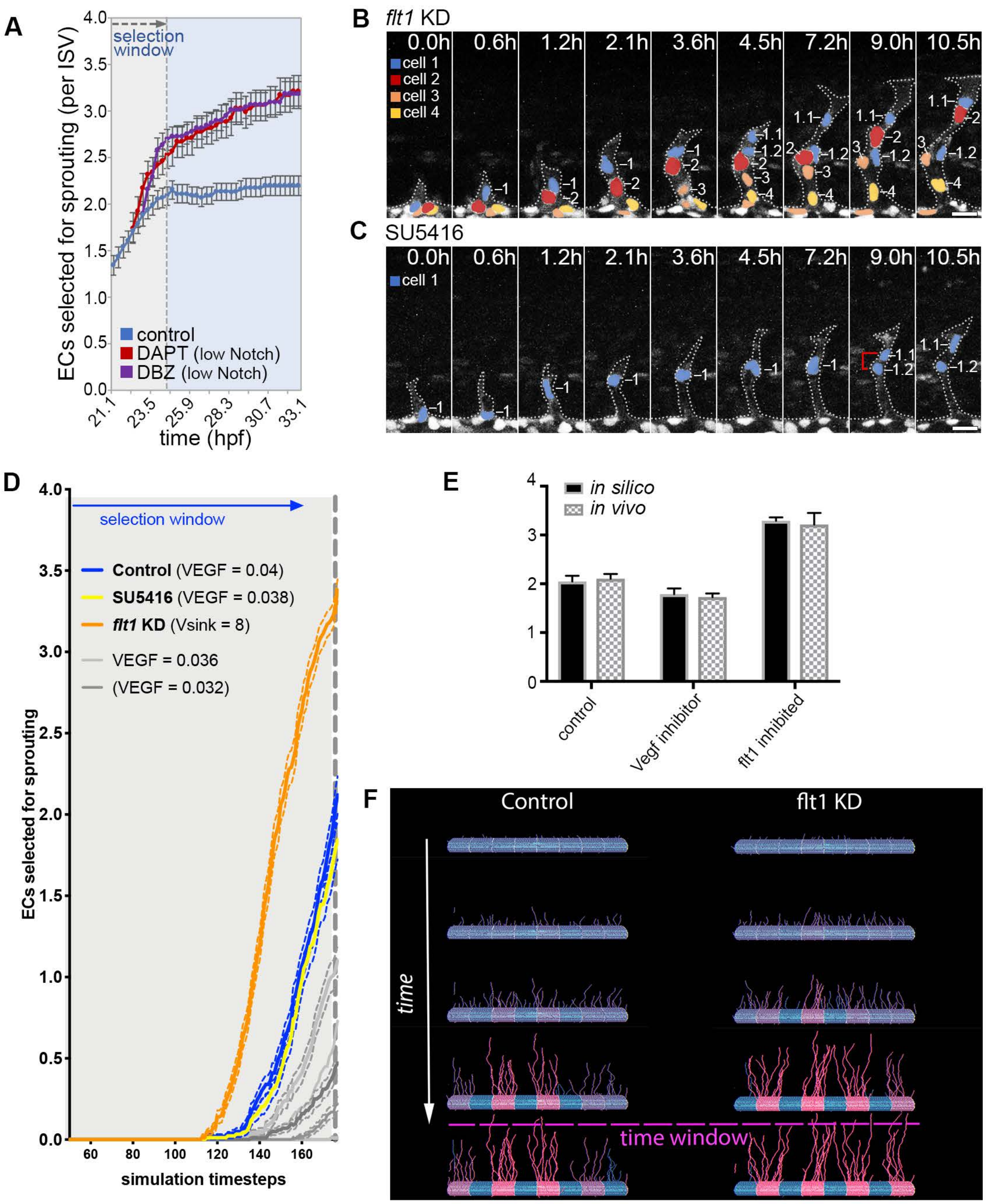

Figure S1. VEGFR signaling and positive-feedback define the magnitude and timing of tip EC selection. Related to Figure 2. (A) Quantification of the number of ECs selected to branch into ISVs of control, DAPT-treated and DBZ-treated embryos (*n* = 47 ISVs from 16 control, 35 ISVs from 8 DBZ-treated and 28 cells from 7 DAPT-treated embryos). (B and C) Time-lapse images of EC nuclei in sprouting ISVs of *flt1* KD (B) and SU5416-treated (C) *Tg(kdrl:nlsEGFP)*^{zf109} embryos from around 19 hours post-fertilization (hpf). Brackets indicate dividing cells. Nuclei are pseudocolored according to when the selected EC entered the ISV. (D) MSM modeling of the influence of differential Vegfr and *flt1* levels on the timing of EC activation via LI. By defining a selection window at approximately 175-time steps, the model accurately recapitulates the *in-vivo* behavior induced by Vegfr inhibition (decreased EC selection) and *flt1* KD (increased EC selection) in Figure 2C. (E) Quantification of the number of cells selected in the selection window both *in-silico* and *in-vivo.* (F) Example frames from one simulation run of a control simulation (Vsink=9) vs a *flt1* KD simulation (Vsink=8) showing by the end of the time window the control is actually less efficient, i.e. slower to select within the time window as the *flt1* KD selects all possible tip cells from the available pool of cells more rapidly. Frames taken at t=0,50,100,150,200. Scale bars, 25 μm.

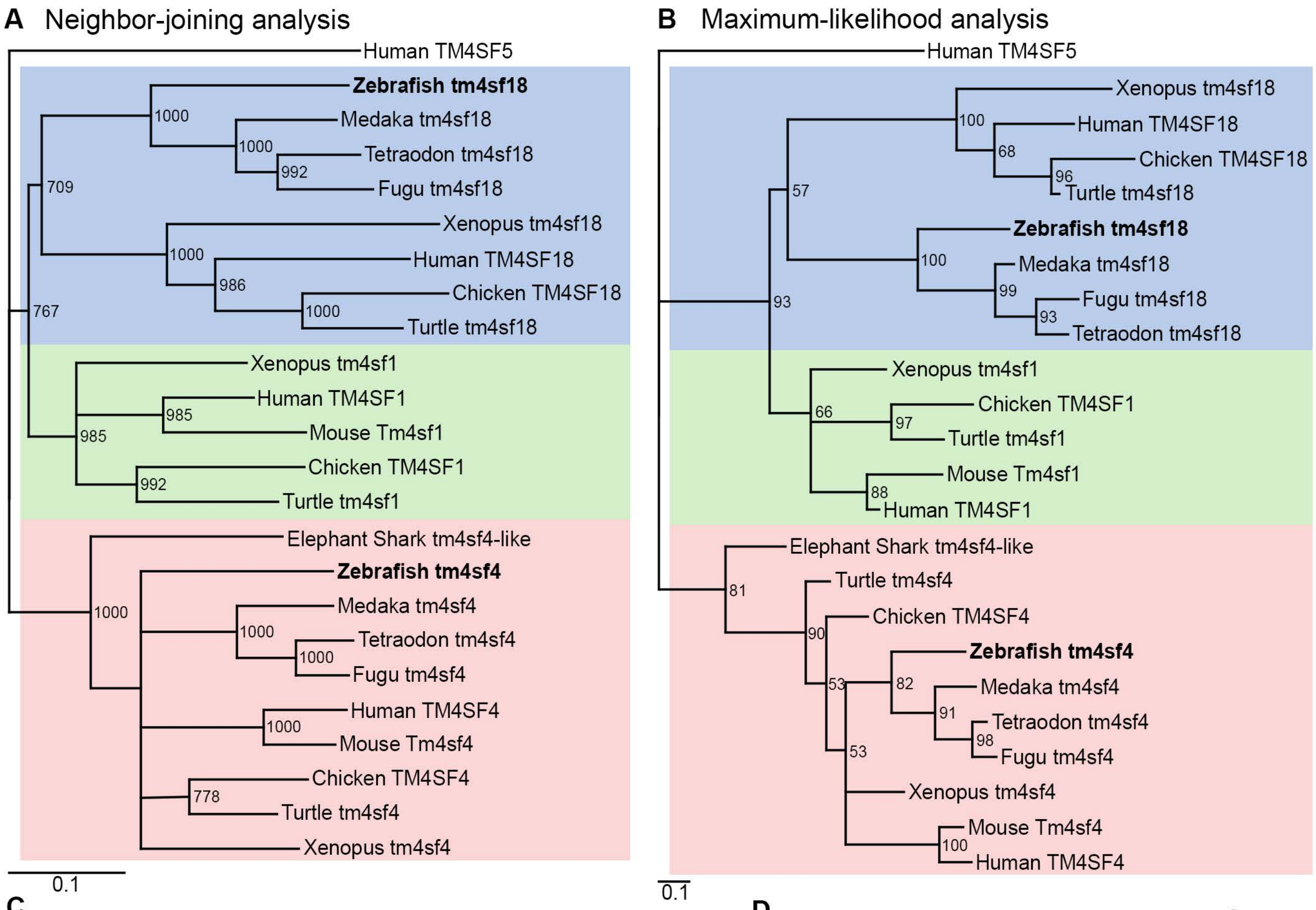

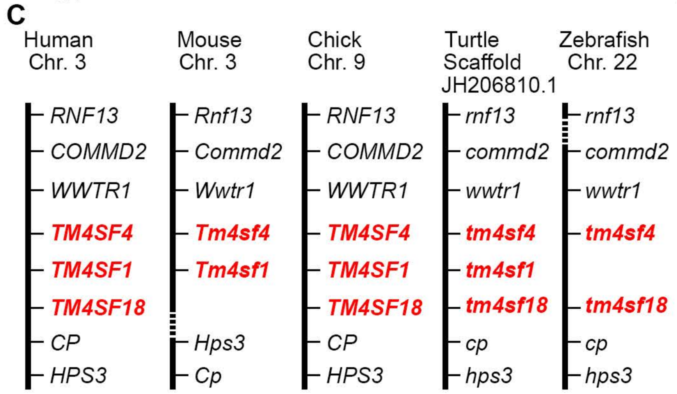

D

	Zebrafish tm4sf18	Medaka tm4sf18	Tetraodon tm4sf18	Fugu tm4sf18
Human TM4SF1	51/66	51/69	45/62	47/63
Human TM4SF18	41/60	39/59	38/56	38/59
Mouse Tm4sf1	47/65	49/65	42/60	45/64
Chicken TM4SF1	47/64	50/64	47/60	47/62
Chicken TM4SF18	40/59	37/56	38/54	37/55

Figure S2. Phylogenetic, synteny and protein analysis of the TM4SF family. Related to Figure 3. (A and B) Phylogenetic trees of vertebrate TM4SF1/4/18 protein family based on either neighbor-joining (NJ, A) or maximum-likelihood (ML, B) analyses. Human TM4SF5 protein sequence was defined as the out-group. Branch lengths are proportional to evolutionary distance corrected for multiple substitutions; the scale bar denotes 0.1 underlying amino acid substitutions per site. Figures on branches indicate robustness of each node (>50%), estimated from 1000 bootstrap replicates for NJ (A) and 100 replicates for ML (B). All nodes with a bootstrap support of less than 50% were collapsed to a polytomy. Based on separate analyses of all vertebrate TM4SF protein (data not shown), TM4SF1, 4 and 18 form a monophyletic group and likely share a common ancestor. Hence, TM4SF1 and TM4SF18 are the most related in the TM4SF family. (C) Synteny analysis of vertebrate *Tm4SF1/4/18* in the human, mouse, chick, turtle and zebrafish genome assemblies. Dashed lines represent breaks in synteny. *TM4SF1, 4* and *18* are closely located on the same chromosome, suggesting that they were generated by two tandem gene duplications at early stages of vertebrate evolution. The first of these gave rise to the *TM4SF4* and *TM4SF1/18* paralogous groups, and the second gave rise to the *TM4SF1* and *TM4SF18* paralogous groups. The *tm4sf1* subfamily appears to have subsequently been lost in the lineage leading to teleost fish. Similarly, the *Tm4sf18* subfamily appears to have subsequently been lost in mice. (D) Percentage BLAST sequence identity (black numbers) and sequence similarity (red numbers) between teleost fish (zebrafish, medaka, tetraodon and fugu) Tm4sf18 and human, mouse or chick TM4SF1/18. Following the loss of *tm4sf1*, the remaining Tm4sf18 in teleost fish consistently shares higher protein sequence identity/similarity with human and chicken TM4SF1 over TM4SF18. Hence, zebrafish Tm4sf18 appears to have acquired the function of Tm4sf1 and is the predominant teleost homologue of mammalian TM4SF1.

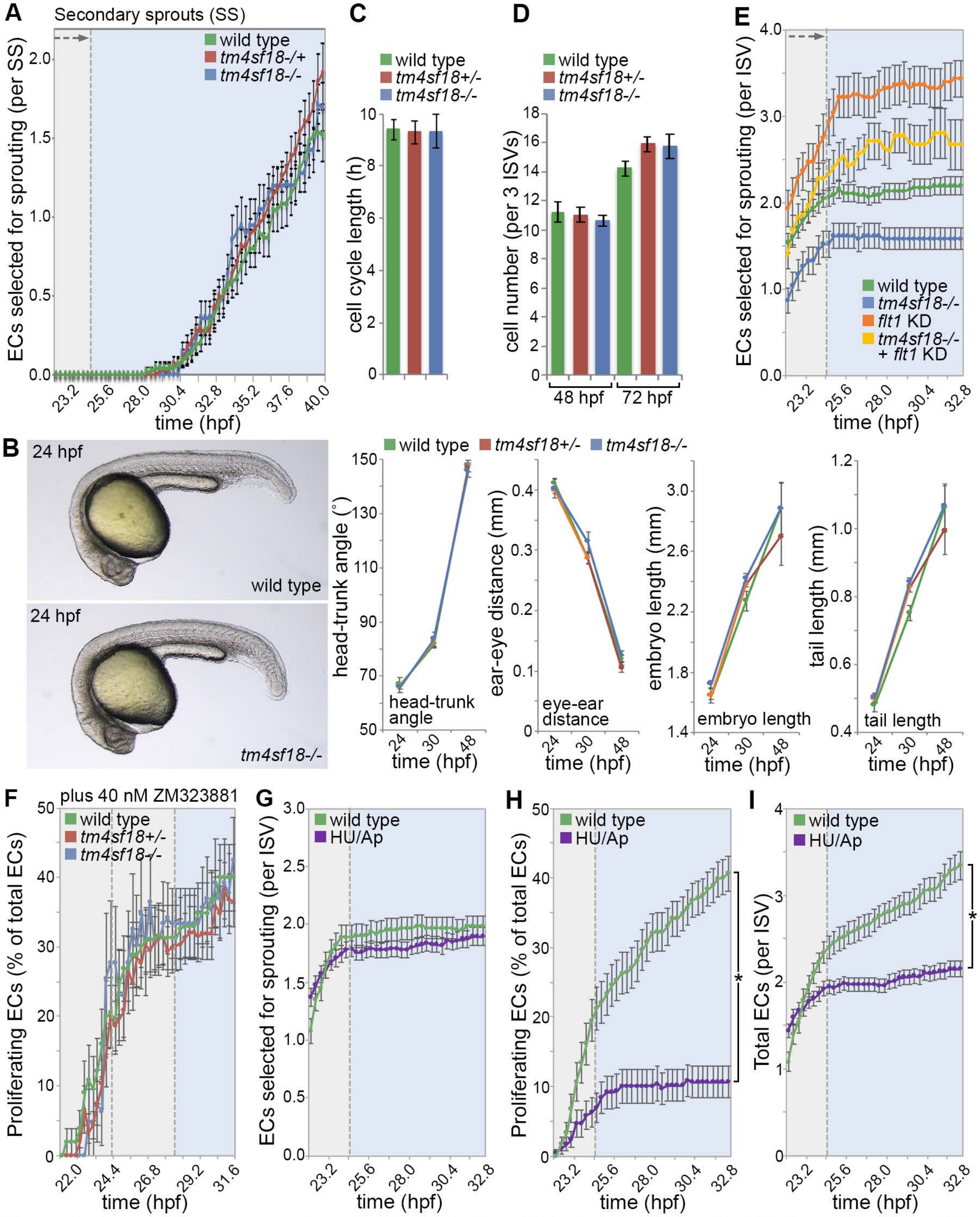

Figure S3. Phenotypic consequences of *tm4sf18* mutation. Related to Figure 5. (A) Quantification of the number of ECs selected to branch into venous secondary sprouts (SS) of WT, *tm4sf18*$^{+/-}$ and *tm4sf18*$^{-/-}$ embryos (*n* = at least 21 ISVs from 16 WT, 12 ISVs from 15 *tm4sf18*$^{+/-}$ and 10 ISVs from 8 *tm4sf18*$^{-/-}$ embryos at each time point). (B) Lateral views of 24 hpf WT and *tm4sf18*$^{-/-}$ homozygous mutant embryos and quantification of key developmental landmarks (*n* = 15 WT, 43 *tm4sf18*$^{+/-}$ and 12 *tm4sf18*$^{-/-}$ embryos at 24 hpf; *n* = 36 WT, 17 *tm4sf18*$^{+/-}$ and 8 *tm4sf18*$^{-/-}$ embryos at 30 hpf; *n* = 17 WT, 29 *tm4sf18*$^{+/-}$ and 18 *tm4sf18*$^{-/-}$ embryos at 48 hpf). (C to D) Quantification of cell cycle length (C; *n* = 29 divisions from 16 WT, 30 divisions from 15 *tm4sf18*$^{+/-}$ and 13 divisions from 8 *tm4sf18*$^{-/-}$ embryos) and ISV cell numbers at 48 hpf and 72 hpf in WT, *tm4sf18*$^{+/-}$ and *tm4sf18*$^{-/-}$ embryos (D; *n* = 18 WT, 38 *tm4sf18*$^{+/-}$ and 25 *tm4sf18*$^{-/-}$ embryos at 48 hpf; *n* = 18 WT, 39 *tm4sf18*$^{+/-}$ and 22 *tm4sf18*$^{-/-}$ embryos at 72 hpf). (E) Quantification of the number of ECs selected to branch into ISVs of WT, *tm4sf18*$^{-/-}$, *flt1* KD and combined *flt1* KD *tm4sf18*$^{-/-}$ embryos (*n* = 47 ISVs from 16 WT, 31 ISVs from 8 *tm4sf18*$^{-/-}$, 28 ISVs from 8 *flt1* KD and from 23 to 9 ISVs from 4 *tm4sf18*$^{-/-}$ *flt1* KD embryos). (F) Quantification of the percentage of ECs that undergo proliferation in WT, *tm4sf18*$^{+/-}$ and *tm4sf18*$^{-/-}$ embryos incubated with 40 nM ZM323881 from 18 hpf onward (*n* = 26 ISVs from 8 WT, 50 ISVs from 14 *tm4sf18*$^{+/-}$ and 21 ISVs from 6 *tm4sf18*$^{-/-}$ embryos). (G) Quantification of the number of ECs selected to branch into ISVs of WT or HU/Ap-treated embryos. (H) Quantification of the percentage of ECs that undergo proliferation in ISVs of WT or HU/Ap-treated embryos. (I) Quantification of the total number of ECs per ISV of WT or HU/Ap-treated embryos (*n* = 62 ISVs from 16 WT and 73 ISVs from 19 HU/Ap-treated embryos). Error bars: mean ± SEM. *$P<0.05$, two-way ANOVA or two-tailed t test.

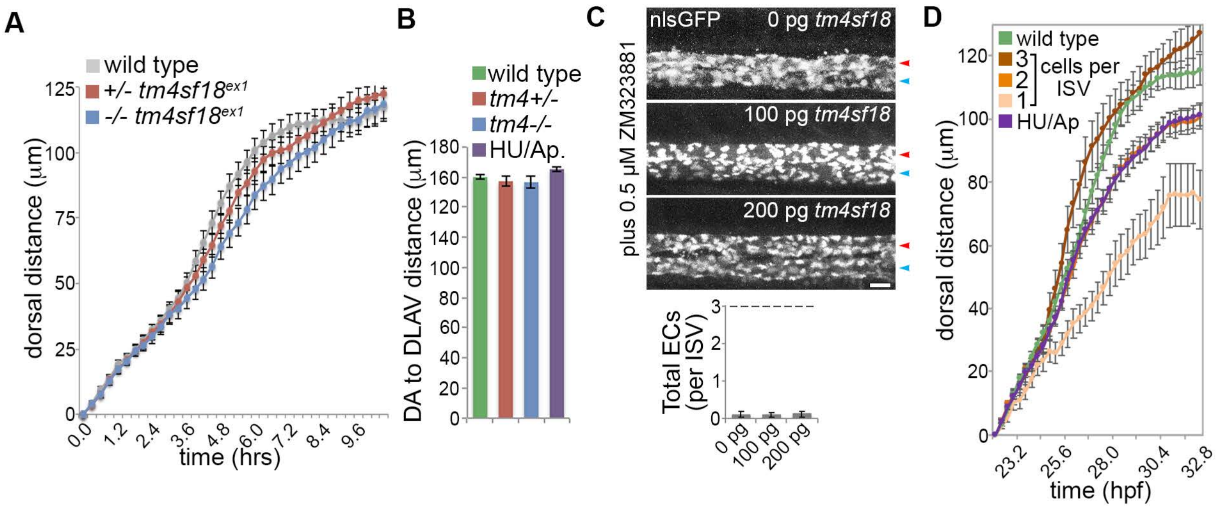

Figure S4. Phenotypic consequences of *tm4sf18* mutation on EC migration. Related to Figure 6. (A) Quantification of the dorsal movement of tip ECs in WT, *tm4sf18*$^{+/-}$ exon1 heterozygous mutant and *tm4sf18*$^{-/-}$ exon-1 homozygous mutant embryos. Mutation of the long (but not short) isoform of *tm4sf18* does not disrupt EC motility (*n* = 71 ISVs from 16 WT, 57 ISVs from 15 *tm4sf18*$^{+/-}$ exon-1 and 38 ISVs from 12 *tm4sf18*$^{-/-}$ exon-1 embryos). (B) Quantification of the length of any fully formed ISVs observed in WT, *tm4sf18*$^{+/-}$, *tm4sf18*$^{-/-}$ and HU/Ap-treated embryos. None of these perturbations affects the morphology of successfully formed ISVs (*n* = 48 ISVs from 16 WT, 45 ISVs from 15 *tm4sf18*$^{+/-}$, 24 ISVs from 8 *tm4sf18*$^{-/-}$ and 66 ISVs from 22 HU/Ap-treated embryos). (C) Lateral views of *Tg(kdrl:nlsEGFP)*^{zf109} embryos at 30 hpf and quantification of total ECs per ISV following injection at the 1 cell stage with the indicated amount of *tm4sf18* mRNA and incubation with ZM323881 from 22 hpf. *tm4sf18* expression is insufficient to drive ISV sprouting in the absence of VEGFR activity. Red and blue arrowheads indicate the DA and PCV, respectively. Dashed line indicates the expected number of ECs per ISV at 30 hpf (*n* = 44 0 pg-injected, 44 100 pg-injected and 40 200 pg-injected embryos). (D) Quantification of the dorsal movement of tip ECs in ISV consisting of 1, 2 and 3 or more ECs and comparison with the motility of tip ECs in wild type and HU/Ap-treated embryos. Tip ECs in wild type embryos behave like those in ISVs with three cells whereas inhibition of proliferation generates tip ECs that behave like those in ISVs with only two cells, consistent with the average number of ECs per ISV in both of these situations (see Figure 5C; *n* = 71 ISVs from 16 WT and 89 ISVs from 22 HU/Ap-treated, as well as 17 ISVs with 3 cells, 53 ISVs with 2 cells and 16 ISVs with 1 cell from 22 embryos). Error bars: mean ± SEM. Scale bar, 25 μm.

Table S1. Primers used for quantitative real-time PCR (qPCR) and genotyping. Related to the STAR Methods.

Experiment	Species	Primer Name	Sequence
qPCR	zebrafish	*β actin* forward	5’-CGAGCTGTCTTCCCATCCA-3’
	zebrafish	*β actin* reverse	5’-TCACCAACGTAGCTGTCTTTCTG-3’
	zebrafish	*dll4* forward	5’-TGGCCAGTTATCCTGTCTCC-3’
	zebrafish	*dll4* reverse	5’-CTCACTGCATCCCTCCAGAC-3’
	zebrafish	*ef1α* forward	5’-CTGGAGGCCAGCTCAAACAT-3’
	zebrafish	*ef1α* reverse	5’-ATCAAGAAGAGTAGTACCGCTAGCATTAC-3’
	zebrafish	*flt4* forward	5’-CTGTCGGATTTGGATTGGGA-3’
	zebrafish	*flt4* reverse	5’-GGTGGACTCATAGAAAACCCATTC-3’
	zebrafish	*kdrl* forward	5’-ACTTTGAGTGGGAGTTTCATAAGGA-3’
	zebrafish	*kdrl* reverse	5’-TTGGACCGGTGTGGTGCTA-3’
	zebrafish	*tm4sf18* forward	5’-CTGGATACTGCTTCCTGATCTC-3’
	zebrafish	*tm4sf18* reverse	5’-CAAACAGATACCGTCCCTCAT-3’
	human	*GAPDH* forward	5’-TGCACCACCAACTGCTTAGC-3’
	human	*GAPDH* reverse	5’-GGCATGGACTGTGGTCATGAG-3’
	human	*TM4SF1* forward	5’-CTTCGTGTGGTTCTTTTCTG-3’
	human	*TM4SF1* reverse	5’-ATCGTTTGCCACAGTTTTC-3’
Genotyping	zebrafish	*tm4sf18* TALEN forward	5’-CTGTTTTCTCCCCCACACAC-3’
	zebrafish	*tm4sf18* TALEN reverse	5’-TACTCACAGCCAGACCACCA-3’
	zebrafish	*tm4sf18* CRISPR forward	5’- CATCAGTCTTTGCAGCGAGA -3’
	zebrafish	*tm4sf18* CRISPR reverse	5’- TGTAGCATATCCCAACACTCAC -3’
